# National-scale mapping of building height using Sentinel-1 and Sentinel-2 time series

**DOI:** 10.1016/j.rse.2020.112128

**Published:** 2021-01

**Authors:** David Frantz, Franz Schug, Akpona Okujeni, Claudio Navacchi, Wolfgang Wagner, Sebastian van der Linden, Patrick Hostert

**Affiliations:** a Earth Observation Lab, Geography Department, Humboldt-Universität zu Berlin, Unter den Linden 6, 10099 Berlin, Germany; b Integrated Research Institute on Transformations of Human Environment Systems (IRI THESys), Humboldt-Universität zu Berlin, Unter den Linden 6, 10099 Berlin, Germany; c Department of Geodesy and Geoinformation, TU Wien, Wiedner Hauptstraße 8/E120, 1040 Vienna, Austria; d Institute of Geography and Geology, University of Greifswald, Friedrich-Ludwig-Jahn-Str. 16, 17489 Greifswald, Germany

**Keywords:** Germany, Machine learning, Copernicus, SAR, Optical, Multi-sensor, Data synergy, Urbanization, Quantitative remote sensing

## Abstract

Urban areas and their vertical characteristics have a manifold and far-reaching impact on our environment. However, openly accessible information at high spatial resolution is still missing at large for complete countries or regions. In this study, we combined Sentinel-1A/B and Sentinel-2A/B time series to map building heights for entire Germany on a 10 m grid resolving built-up structures in rural and urban contexts. We utilized information from the spectral/polarization, temporal and spatial dimensions by combining band-wise temporal aggregation statistics with morphological metrics. We trained machine learning regression models with highly accurate building height information from several 3D building models. The novelty of this method lies in the very fine resolution yet large spatial extent to which it can be applied, as well as in the use of building shadows in optical imagery. Results indicate that both radar-only and optical-only models can be used to predict building height, but the synergistic combination of both data sources leads to superior results. When testing the model against independent datasets, very consistent performance was achieved (frequency-weighted RMSE of 2.9 m to 3.5 m), which suggests that the prediction of the most frequently occurring buildings was robust. The average building height varies considerably across Germany with lower buildings in Eastern and South-Eastern Germany and taller ones along the highly urbanized areas in Western Germany. We emphasize the straightforward applicability of this approach on the national scale. It mostly relies on freely available satellite imagery and open source software, which potentially permit frequent update cycles and cost-effective mapping that may be relevant for a plethora of different applications, e.g. physical analysis of structural features or mapping society's resource usage.

## Highlights

• We present a method to predict building height on a 10 m grid for entire Germany.
• Synergistic use of VV/VH Sentinel-1A/B and multi-spectral Sentinel-2A/B time series
• Training and rigorous validation with high-quality 3D Building Models
• Building height predicted with a frequency-weighted RMSE of 3.2 m to 4.2 m.
• Mean building height in Germany is correlated with population density.

## 1 Introduction

Three quarters of humanity currently lives in cities and towns (Dijkstra et al., 2020), and it is estimated that this trend will continue unabatedly. While cities only cover a small portion of the Earth's land surface, their impact is far-ranging as they are, for example, accountable for up to 80% of total energy consumption and 75% of carbon emissions (United Nations, 2020). For understanding urban process regimes, knowing building heights is key (Zhu et al., 2019), and the vertical structure of settlements has been identified as a central parameter to systematize multi-dimensional urban form (Wentz et al., 2018). Many quantities scale linearly with building height. For example, building height – or related information like floor area – has been shown to be an important indicator for estimating energy consumption (Resch et al., 2016), material stock allocation (Tanikawa et al., 2015), greenhouse gas emissions (Borck, 2016), human wellbeing and urban heat island effects (Perini and Magliocco, 2014), or the distribution of population (Alahmadi et al., 2013). The latter is also of increasing relevance as knowledge about the distribution and concentration of the population may help in better understanding the risk of spreading infectious diseases (Wu et al., 2017).

Building height can be derived with a broad variety of geoinformation or remote sensing-based approaches. Nonetheless, it is a parameter that is hard to quantify accurately and at regular intervals for larger areas such as complete countries and at a spatial resolution that approximates individual building footprints. The two-dimensional building footprint from building cadasters can be extruded to three-dimensional cubes (often referred to as “Level-of-Detail 1” or LoD1), if databases include attributes like the number of floors, floor height or building function (Biljecki et al., 2017). The combination of cadastral building footprints with airborne laser scanning (ALS) represents an elaborated extension of the latter approach. This technique has become the quasi-standard for generating 3D building models at LoD2, wherein prototypical roof shapes are derived from the actual point cloud (Aringer and Roschlaub, 2014). Although open data policies are increasingly emerging in general (Nugroho Rininta et al., 2015), many datasets are still proprietary and come with considerable data purchase costs (e.g. Bayerische Vermessungsverwaltung, 2020). Open alternatives to cadastral data are mostly community-based (e.g. OpenStreetMap) and as such are regionally inconsistent (Neis et al., 2013) and spatially incomplete (Brovelli and Zamboni, 2018; Hecht et al., 2013). Alternatively, building height can be measured in very high resolution (VHR) images by (i) measuring the building's cast shadows in optical images (Wang et al., 2014), (ii) by photogrammetrically analyzing stereo pairs of optical satellite, aerial or drone images (Stal et al., 2013; Takaku et al., 2016; Unger et al., 2014), (iii) or by interferometric synthetic aperture radar (InSAR) techniques (Stilla et al., 2003). However, measuring shadows is less reliable when buildings shadows overlap each other (Biljecki et al., 2017), in which case also the SAR side-looking geometry causes adverse layover and shadow effects (Stilla et al., 2003). Photogrammetric height retrievals from aerial images are more accurate than their space-based counterparts (Sirmacek et al., 2012), which is related to an increasing error with flying altitude (Baltsavias, 1999). Although photogrammetry and ALS may be similarly accurate (Baltsavias, 1999), ALS is superior for measuring building height (Kaartinen et al., 2005), especially when paired with 3D city models where the footprints are coming from official cadasters. Notwithstanding, any acquisition technique without a global observation scenario, e.g. ALS, UAV, and airborne or spaceborne VHR imaging alike, ultimately faces challenges regarding actuality, continuity and regional consistency. The same applies for data affected by non-open data policies. For example, TerraSAR-X / TanDEM-X imagery for InSAR processing is freely available, but limited to an area of 100,000 km^2^ and only available for scientists and on request (DLR, 2019).

Eventually, free and open, globally available satellite image archives with standardized and consistent data processing are required to allow building height estimation for large areas at fine resolution. Among the hundreds of EO satellite missions (Belward and Skøien, 2015), only few systems fulfill these requirements. The European Copernicus program (Aschbacher and Milagro-Pérez, 2012) encompasses the Sentinel-1 and Sentinel-2 constellations with a high potential for building height mapping. The Sentinel-1 constellation provides all-weather, day-and-night C-band Synthetic Aperture Radar (SAR) backscatter observations at 10 m spatial gridding (Torres et al., 2012). The primary observation mode over land surfaces is interferometric wide (IW) swath, which covers a 250 km swath at two polarizations (VV and VH). The mission currently comprises two satellites, Sentinel-1A and Sentinel-1B, providing a same-orbit revisit frequency of 6 days. Due to lateral orbit overlaps, the actual revisit frequency increases with latitude; over Europe it is 1–3 days. Li et al. (2020b) have recently shown that Sentinel-1 backscatter is helpful for estimating 3D information in cities when mapping building height as an average elevation per raster cell, i.e. a mean value of building heights and non-built-up surfaces in-between. They exploited the fact that backscatter is in general positively correlated with building height (Koppel et al., 2017), due to specular reflectance by urban structures and a combination of single bounce, double bounce (dihedrals), and triple bounce (trihedrals) scattering mechanism (Dong et al., 1997). However, the relationship between backscatter and height varies with building orientation relative to the sensor, surface material, and roughness (Corbane et al., 2008; Kimura et al., 2005; Koppel et al., 2017; Li et al., 2016). Li et al. (2020b) therefore proposed an index from combined VV- and VH-polarized SAR data and in doing so increased the predictive strength of the statistical relationship with building height. Nevertheless, they report several urban layouts and structures where this relationship is weak or non-existent, including effects related to double bouncing, high reflectivity from metallic materials, as well as scattering related to tree canopy and building density. Therefore, it appears beneficial to add information from optical imagery that allows to complement ambiguous information in the radar signal and improve the signal's correlation with building height.

**Fig. 1:**
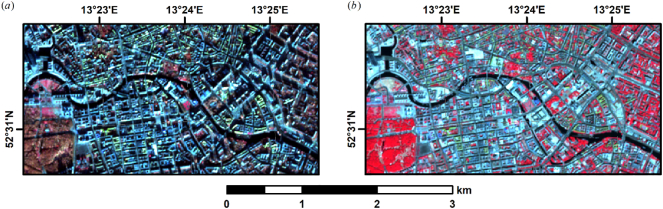
Building-height induced shadows in Sentinel-2 imagery. (a): Sentinel-2A image, 05.12.2018; (b): Sentinel-2B image, 03.07.2018. The images depict the center of Berlin as false colour representation, i.e. R/G/B = near infrared/red/green. (For interpretation of the references to colour in this figure legend, the reader is referred to the web version of this article.)

The Sentinel-2 satellite mission provides multi-spectral optical observations in 13 wavelengths at up to 10 to 60 m spatial resolution (Drusch et al., 2012). The mission comprises Sentinel-2A and Sentinel-2B, which are operated in the same orbit with a phase delay of 180°, providing a nadir revisit frequency of 5 days (Drusch et al., 2012). Due to the wide swaths of 290 km, lateral orbital overlaps can be considerable and partially increase revisit frequency to 2 to 3 days. Similar optical Earth observation missions have already proven their ability to contribute to generating 3D information, e.g. to map forest height with Landsat data (Helmer et al., 2010; Li et al., 2011; Wang et al., 2018). Li et al. (2020a) have recently shown that Landsat and Sentinel-1 (among others) are capable of predicting building height and volume at a coarse 1 km resolution, yet, their feature importance assessment indicates that their model is mostly driven by the radar data. Still, the contrast between roofs and shadows is easily visible in optical winter imagery (Fig. 1a). Taking into account the clear seasonality of this contrast (cf. summer imagery, Fig. 1b), the spatio-temporal information existing in time series of multi-spectral data may increase the value of the reflectance data.

Thus, we hypothesize that the synergistic usage of information from multi-temporal and multi-spectral acquisitions may well complement the capabilities of dual-polarized radar backscatter. In this study, we aim at creating a methodology to predict building height from free, open, and globally available image archives using machine learning regression. We produce a building height map for entire Germany at unprecedented spatial resolution approximating building footprints. For this, we use dual-polarized Sentinel-1 and multi-spectral Sentinel-2 time series, and rely on highly accurate training and validation data derived from 3D building models. We particularly address the following research questions:

• Are synergistic models based on Sentinel-1 radar and optical Sentinel-2 data superior to using radar or optical data alone when predicting building height?
• How accurately can building height be predicted?
• Can the machine learning models be transferred to other regions without significant decrease in prediction accuracy?
• What regional patterns of building height distribution can be observed across our study area?

## 2 Study area

**Fig. 2:**
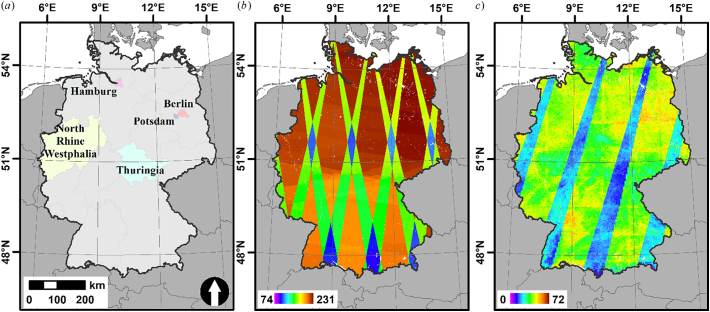
Study area and data availability. (a): study area with locations of training and validation data (colored polygons); (b): number of Sentinel-1A/B acquisitions for 2017; (c):number of clear-sky, non-snow Sentinel-2A/B observations for 2018.

Our region of interest for this study was Germany, covering a total area of about 357,000 km^2^ (Fig. 2a). Germany has about 83.1 Mio. inhabitants; population density is 232 cap km^−2^, varying from 69 cap km^−2^ to 4090 cap km^−2^ in the federal states (Statistisches Bundesamt (Destatis), 2020). About 9.6% of the area is covered by built-up infrastructure (Schug et al., 2020). The majority of buildings are residential (19,053,216 in 2018), half of them in the states of North Rhine Westphalia, Baden-Württemberg and Bavaria (Statistisches Bundesamt (Destatis), 2019b). The majority of these houses are single-family houses (82% one or two dwelling units); only 6% are tall multi-family houses (>7 dwellings), however they make up for a third of all German dwellings (Landesamt für Statistik Niedersachsen, 2014). Information about the non-residential building stock is less reliable and conclusive; it is estimated that there are about 3 Mio. non-residential buildings (Deutsche Energie-Agentur (dena), 2016).

## 3 Data

### 3.1 Independent variables

#### 3.1.1 Sentinel-1

Sentinel-1 backscatter data for the year 2017 were accessed from the data archive of the Earth Observation Data Centre (EODC, Wagner et al., 2012). The EODC infrastructure couples an OpenStack cloud platform and the supercomputing resources of the Vienna Scientific Cluster (VSC) with a Petabyte-scale storage that contains among other satellite data the complete worldwide Sentinel-1 IW swath data record. The EODC data archive has been built up by continuously downloading Sentinel data from the Copernicus data hubs. To ensure the completeness of the data collection and short data latencies, a “Hubwatcher” is deployed to monitor and cross-check several Copernicus data hubs. In this study we used ground range detected (GRD) and high-resolution (HR) IW scenes, which contain backscatter intensities at 10 m pixel spacing in VV or VH polarization for 2017. As suggested by Koppel et al. (2017), Sentinel-1 data from both orbit directions were combined to enlarge the observation space for maximization of information content and reduction of areas in radar shadows. To allow efficient spatio-temporal analysis of the Sentinel-1 IW swath data, we pre-processed the original Level-1 data to generate a Sentinel-1 data cube based upon the Equi7 grid (https://github.com/TUW-GEO/Equi7Grid) developed by (Bauer-Marschallinger et al., 2014). The pre-processing was orchestrated and carried out using the SAR Geophysical Retrieval Toolbox (SGRT) developed by TU Wien, whereas the Sentinel Application Platform (SNAP) and the freely available 3-arc *sec* SRTM terrain model (Farr et al., 2007) were used for radiometric calibration and Range Doppler geometric terrain correction. Border noise effects, which affect a certain percentage of the original Sentinel-1 IW scenes, were eliminated with the bidirectional all-samples approach introduced by (Ali et al., 2018). The so-generated Sentinel-1 data cube holds backscatter values (sigma-naught in dB) sampled regularly at 10 m in space and irregularly in time (corresponding to the exact times of the Sentinel-1 data takes) along with local incidence angles and other relevant metadata.

Fig. 2b shows data availability; overlapping orbits and looking directions cover a large part of the study area and increase nominal revisit frequency to 1–2 days for a substantial part of the study area.

In a final post-processing step, all images were reprojected to EPSG:3035, and then split to 30 × 30 km data cubes (Frantz, 2019; Lewis et al., 2016) to match the characteristics of the optical data (see next subsection).

#### 3.1.2 Sentinel-2

We downloaded all Sentinel-2 Level 1C products acquired in 2018 with cloud coverage <70% from the ESA API Hub. The Sentinel-2 constellation was fully ramped up on 17 February 2018, thus providing full data density since this date (ESA, 2018). The study area is covered by 69 Military Grid Reference System (MGRS) tiles, and a total number of 7278 image products were available. Fig. 2c shows clear-sky data availability, where lateral orbital overlaps cover a large part of the study area and partially increase nominal revisit frequency to 2–3 days.

The data were processed to Level 2 Analysis Ready Data (ARD) through the Framework for Operational Radiometric Correction for Environmental monitoring (FORCE, Frantz, 2019) available from https://github.com/davidfrantz/force. Clouds and cloud shadows were identified using a modified version of the Fmask algorithm (Frantz et al., 2016a; Frantz et al., 2015; Zhu et al., 2015; Zhu and Woodcock, 2012), including a parallax-based extension to better separate clouds from bright targets such as built-up structures (Frantz et al., 2018). All images were radiometrically standardized as outlined by Frantz et al. (2016a). The atmospheric correction included radiative transfer modelling (Tanré et al., 1979), object-based Aerosol Optical Depth (AOD) estimation over dense dark vegetation (Kaufman and Sendra, 1988) and water (Royer et al., 1988), topographic correction using an enhanced C-correction as described in Buchner et al. (2020), adjacency effect correction (Bach, 1995), and Nadir BRDF-adjustment using a global set of MODIS-derived BRDF kernel parameters (Roy et al., 2016). Surface reflectance retrieval, as well as AOD estimation and cloud masking were assessed in the Atmospheric Correction and Cloud Masking Inter-comparison eXercises (ACIX/ACIX-II/CMIX, Doxani et al., 2018; ESA, 2019). The 20 m bands were improved to 10 m resolution using a data fusion approach, (Frantz et al., 2016b), wherein 10 m information from the local pixel neighborhood is used to weight the 20 m pixels using a weighted average approach. The 60 m bands were discarded. Eventually, all images were reprojected to a single coordinate system (EPSG:3035), and then split to 30 × 30 km image chips to form data cubes (Frantz, 2019; Lewis et al., 2016).

### 3.2 Dependent variable: building height

For building height training and validation, we used freely and openly available 3D building models (3DBM) in CityGML standard format. 3DBMs provided by geodetic surveys are optimal input for our purpose, as they are high quality compilations of multiple data sources. The building height information is typically based on official ALS campaigns of the local geodetic surveys, and is merged with building footprints from the official cadaster. Thus, in contrast to ALS point clouds, height information is provided for buildings only; no further disentanglement between buildings and other vertical structures was necessary (as e.g. required by the approaches of Geiß et al. (2019) and Esch et al. (2020)). As outlined in the introduction however, 3DBMs alone are insufficient to produce nation-wide building height maps, as they are only available for limited areas, where local administrations have decided on open data policies. We have acquired five 3DBMs, which cover entire cities to states (Table 1) and include nearly 15 million buildings. Those are mostly available as LoD1 or LoD2. In LoD1, each building is a block without considering its actual roof shape (accuracy of building height typically ±5 m); LoD2 additionally contains a standard roof shape that best fits to the LiDAR point cloud (accuracy of building height typically ±1 m). Due to the better accuracy, we only used LoD2 models in this study. The datasets were acquired between 2012 (Potsdam) and 2020 (Thuringia) with some within-dataset inconsistency (Table 1). Due to the fairly low net changes in the German building stock (Statistisches Bundesamt (Destatis), 2019a), we consider these temporal differences negligible given the purpose of this study.

**Table 1:** 3D Building Models used for training and validation.

Site	Year	Accuracy in height^1^	Buildings	Data provider	License	Link
Berlin	2014 (99.9%) 2015 (0.1%)	unknown	540,172	Berlin Partner für Wirtschaft und Technologie GmbH	Custom license: https://www.businesslocationcenter.de/berlin3d-downloadportal/documents/terms.en.html	https://www.businesslocationcenter.de/en/economic-atlas/download-portal/
Hamburg	2016	±1 m	374,990	Freie und Hansestadt Hamburg, Landesbetrieb Geoinformation und Vermessung	Data license Germany – attribution – version 2.0	http://suche.transparenz.hamburg.de/dataset/3d-stadtmodell-lod2-de-hamburg4?forceWeb=true
Potsdam	2012	unknown	44,832	Landeshauptstadt Potsdam (LHP)	Unspecified open data license	https://opendata.potsdam.de/explore/dataset/3d-gebaudemodell-lod2-citygml/information
North Rhine Westphalia	2018 (39%) 2019 (61%)	±1 m	11,498,734	Bezirksregierung Köln, Geobasis NRW	Data license Germany – attribution – version 2.0	https://www.bezreg-koeln.nrw.de/brk_internet/geobasis/3d_gebaeudemodelle/index.html
Thuringia	2018 (13%) 2019 (57%) 2020 (30%)	unknown	2,241,792	Kompetenzzentrum Geodateninfrastruktur Thüringen (GDI-Th)	Data license Germany – attribution – version 2.0	https://www.geoportal-th.de/de-de/Downloadbereiche/Download-Offene-Geodaten-Th%C3%BCringen/Download-3D-Geb%C3%A4ude

1 Accuracy according to data publisher; errors may be larger for more complex roof types.

### 3.3 Other data

Similar to Li et al. (2020b) and Li et al. (2020a), we masked our final building height map to areas covered by residential and non-residential buildings only. Note that this was performed after the statistical validation. We used the European Settlement Map (ESM, Corbane and Sabo, 2019; Sabo et al., 2019), which is part of the Global Human Settlement Layer (GHSL) datasets made available by the European Commission (https://ghsl.jrc.ec.europa.eu/data.php). The dataset is based on Copernicus VHR imagery for the reference year 2015, and is generated at 2 m spatial resolution from Pleiades, Deimos-02, WorldView-2, WorldView-3, GeoEye-01 and Spot 6/7 images, which were acquired between 2014 and 2016. As net change of building stock is positive (+114,543 new buildings in 2018 (Statistisches Bundesamt (Destatis), 2019a)), our final map is representative for ca. 2015.

## 4 Methods

### 4.1 Theoretical background

**Fig. 3:**
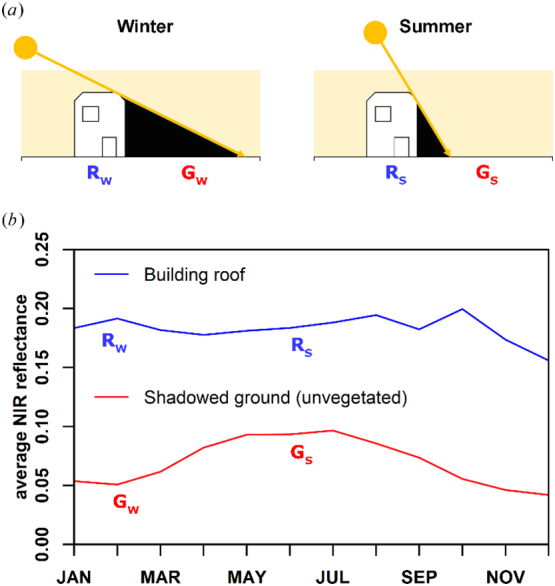
Temporal effect of building shadows in Sentinel-2 imagery. (a): schematic representation of the shadowing effect for winter (left, subscript W) and summer (right, subscript S). (b): monthly near infrared average of all available clear-sky observations for a building roof pixel (blue), and the ground that is shadowed by this building (red); the two pixels were drawn from the subset shown in Fig. 1. (For interpretation of the references to colour in this figure legend, the reader is referred to the web version of this article.)

As illustrated by Fig. 1, winter imagery in sun-synchronous optical imagery is much affected by shadows from vertical objects (e.g. buildings), whereas only the tallest objects cast a discernible shadow in summer imagery from Germany. In addition, there is also a distinct seasonal pattern in reflectance. Due to the constant overpass time of Sentinel-2, the sun elevation progressively increases towards the summer solstice, which results in decreasing building shadow lengths (Fig. 3a), which in turn results in a progressive increase of reflectance for flat areas next to buildings at 10 m spatial resolution (Fig. 3b, red line). Roof pixels that are sunlit throughout the entire year do not show this seasonal pattern (Fig. 3b, blue line).

**Fig. 4:**
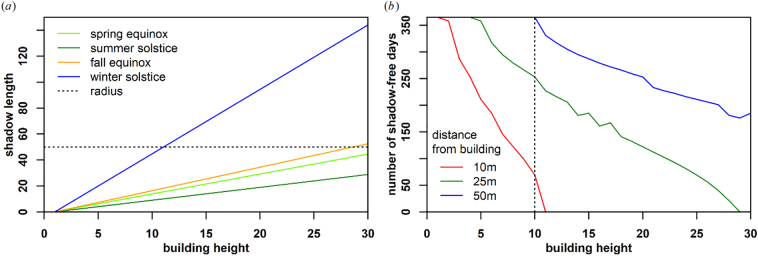
Computations of building shadow lengths. (a): building shadow length in dependence on season, shown for four key astronomical dates. (b): number of shadow-free days when being 10 m, 25 m, or 50 m away from buildings with different height. The figure reads like this: for a 10 m high building (vertical line), we have 69, 253 and 365 shadow-free days a year if we step 10 m, 25 m, or 50 m away from the building.

These findings are further corroborated by the calculations shown in Fig. 4. Given a 10:30 local time overpass for 52.51°N (e.g. Berlin, Germany), the building shadow length *d* can be computed with

(1) d=h/tanα,

where *h* is the building height and *α* the sun elevation (Bivand and Lewin-Koh, 2019). For a large part of the year, i.e. between spring and fall equinox, which also coincides with highest optical data availability, shadow lengths for even tall buildings are usually below 50 m. Fig. 4b additionally demonstrates that the spatio-temporal effect found in Fig. 3b is also dependent on building height. In dependence of building height, the theoretical figure shows the number of days we are observing a shadow-free ground when being 10 m, 25 m, or 50 m away from the building.

### 4.2 Independent variables

Considering the theoretical background, we consequently developed a workflow that (1) uses radar backscatter and spectral reflectance, and (2) synergistically includes spatial context, multi-temporal, multi-spectral, and multi-polarized information. We generated an extensive feature space from the Sentinel-1 and Sentinel-2 time series, which include spectral and temporal information (subsection 1), as well as additional spatial information in a second step (subsection 2). Both steps were performed using FORCE (https://github.com/davidfrantz/force). For the sake of simplicity, we refer to “spectral” information throughout the remainder of the paper, although acknowledging that this is technically incorrect to some degree as e.g. different radar polarizations are measured at the same wavelength.

#### 4.2.1 Spectral-temporal features

As we identified temporal patterns in the satellite data, which are dependent on building height, we generated Spectral Temporal Metrics (STM, Mack et al., 2017; Müller et al., 2015; Potapov et al., 2009). These are statistical aggregations of all available high-quality observations within a specified time period (one year in this case), and as such provide rich information on spectral-temporal variability and data distribution (Frantz, 2019). Due to their spatial completeness and fairly high robustness against different observation densities, they are optimal features for machine learning applications, and have proven to be effective for a large variety of land cover mapping or quantitative variable estimation (e.g. Rufin et al., 2019; Schug et al., 2020). For the optical time series, only clear-sky, non-cloud, non-cloud shadow, non-snow observations were considered. For the radar time series, each observation was considered. All statistics available in FORCE (Frantz, 2019) were used: average, standard deviation, 0/10/25/50/75/90/100% quantiles, range, interquartile range, skewness, and kurtosis. The STMs were generated separately for each band of the SAR (2 bands, i.e. VV and VH polarization) and optical time series (10 land application bands covering VIS, NIR and SWIR domains). Additionally, six spectral indices derived from the optical time series were selected. The Normalized Difference Vegetation Index (NDVI, Tucker, 1979) and Tasseled Cap Greenness (Crist, 1985) were selected to account for urban vegetation, which was e.g. problematic in the Sentinel-1-only study by Li et al. (2020b); two “green” indices were chosen because NDVI alone is less suitable in Central Business Districts where shadow is dominating. The Normalized Difference Built-Up Index (NDBI, Zha et al., 2003) was used due to its sensitivity to urban areas. The Tasseled Cap Brightness (Crist, 1985) was chosen to capture brightness gradients of roofing materials. The Tasseled Cap Wetness (Crist, 1985) and modified Normalized Difference Water Index (mNDWI, Xu, 2006) were chosen to potentially account for water intermingled within the settlement, and as they emphasize shadows. The combination of 18 bands/indices and 13 statistical aggregations resulted in 234 spectral-temporal features.

#### 4.2.2 Spatial-spectral-temporal features

To capture the spatial characteristics originating from shadows cast off from nearby buildings, we computed texture metrics on top of the STMs, denoted as spatial STM (SSTM). We used morphological metrics as they are a standard way to capture spatial context in image processing (Mura et al., 2010), and can e.g. be used to extract the darkest pixel within a pixel's neighborhood. We computed erosion, dilation, opening, closing, morphological gradient, top hat and black hat using a circular structuring element with a 50 m radius. This radius was chosen as a compromise to capture most shadows (Fig. 4 shows that even taller buildings (30 m) cast a shadow smaller than 50 m for almost the entire year), while not losing too much spatial detail. This procedure resulted in a feature space with 234 × 7 = 1638 features.

### 4.3 Dependent variable: building height

#### 4.3.1 Data extraction

The 3DBMs available to us are of very high quality in terms of location, geometry, and height. However, without further processing, they are no adequate training (or validation) data for building height mapping with decameter resolution EO data. In the CityGML format, buildings are further split into building parts, which are the geometries attributed with a building height value. However, these parts can range from complete buildings to small building extensions or garages. Sampling a garage, which often is located next to a taller building, would lead to undesired results when paired with satellite imagery, in which the garage and building are intermingled in one pixel. In addition, as outlined in the last section, a pure per-pixel estimate of building height would not work as we strive to incorporate surrounding information about the building's shadows in a 50 m radius. As such, all building parts in the local neighborhood need to be taken into account to match the prepared features, which was enabled by the spatial completeness of the official cadaster-based input datasets.

**Fig. 5:**
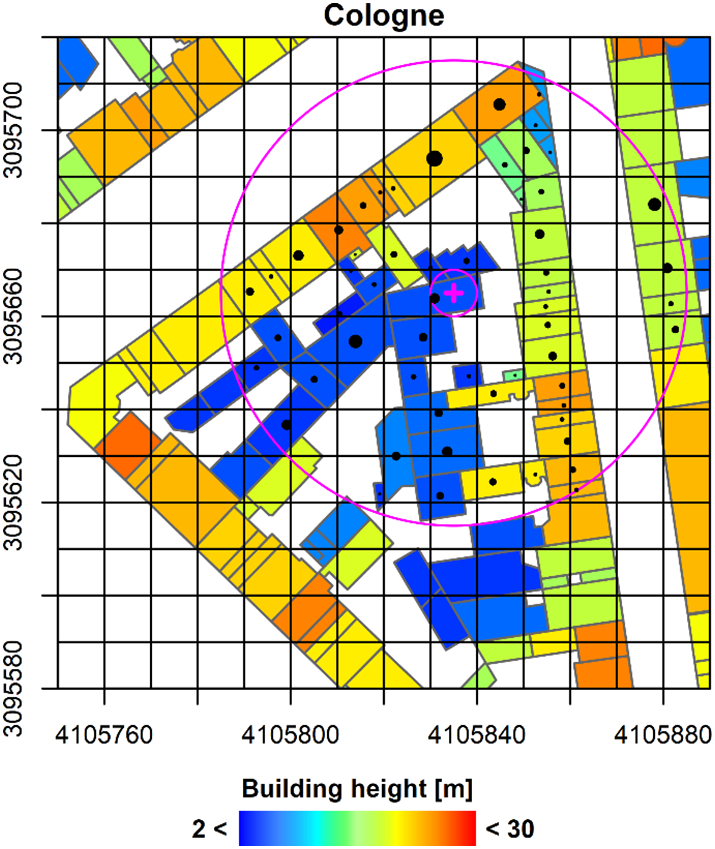
2D representation of a small part of a 3D Building model and basic principle of the sample generation. The footprint of each building part is drawn, and the building height is represented through colour. The black grid represents the pixel grid of the cubed EO images. The pink cross marks the current pixel; pink circles represent 5 m and 50 m buffers. The black points are building part centroids within the 50 m radius; the point size corresponds to the footprint area of the building parts. Coordinate system is EPSG:3035. (For interpretation of the references to colour in this figure legend, the reader is referred to the web version of this article.)

Fig. 5 displays a small portion of the 3DBM for Cologne as 2D representation, as well as the basic principle of our sample preparation. To speed up processing, we first parse the 3DBM, and store the height, footprint area and centroid of each building part in a table. Then, we overlay the 10 m pixel grid of the cubed EO data (black grid). For each pixel, e.g. the one marked with the “+” signature, it is checked whether a building part centroid (point signature) is within 5 m distance (small circle). If yes, all building parts with their centroids closer than 50 m (large circle) are included as

(2) $$\bar{z}=\frac{\sum_{p}^{n}z_{p}∙A_{p}}{\sum_{p}^{n}A_{p}},$$

wherein the area-weighted height $\bar{z}$ is computed from the building height *z*_p_ of all nearby building parts *p*. The weights are the areas of the building parts *A*_p_ (as indicated by different point sizes in Fig. 5). Note that we only average the height of buildings – and not the ground in between as e.g. done by (Li et al., 2020b).

In the sampling process, selected building categories were removed, as we considered them structurally too specific and of rare occurrence, for example power poles of wind energy plants. Table 2 summarizes the number of potential samples per dataset; note that these numbers differ from Table 1 as the latter reports number of buildings, whereas the 3DBM processing is based on building parts.

**Table 2:** Number of retrieved samples per 3D Building Model.

Site	Berlin	Hamburg	Potsdam	North Rhine Westphalia	Thuringia
Number of potential samples	435,610	445,729	36,592	7,651,841	960,712
share of one to two floors (2–6 m)	15.49%	13.83%	22.75%	7.92%	5.66%
share of three to five floors (6-15 m)	55.88%	74.37%	66.94%	89.41%	90.77%
share of five to ten floors (15–30 m)	26.56%	11.51%	9.71%	2.62%	3.51%
share of high-rise (30–150 m)	2.07%	0.29%	0.60%	0.05%	0.05%
share of sky-scrapers (> 150 m)	0.0012%	none	none	0.0001%	none
Samples per height slice	1000	1000	1000	1000	1000
Number of retrieved samples	29,559	21,513	10,127	28,995	20,805

#### 4.3.2 Sampling for training and validation

Within each 3DBM, we sampled sufficiently large, yet reasonably small datasets, which were used for training, as well as for hold-out and leave-one out cross-validation setups (see Table 2). This sample was drawn using constrained stratification of the building height: with 1 m resolution, the same number of samples (if available) was taken from all potential samples. For this, the dataset was iteratively sliced into meters, and within each slice, random samples were iteratively drawn. Due to their rare occurrence, all heights above 50 m were included in the 50 m slice to avoid that each and every high-rise building was selected. If the sample candidate was located within 50 m of another confirmed sample, it was discarded. This was repeated until enough samples for each height slice were retrieved, if all potential samples were collected, or if we exceeded 100,000 iterations. In order to avoid a systematic rejection of tall or low buildings, the iterative procedure of height slicing was reversed after each iteration, i.e. in the first iteration samples were taken from the 3 m to 4 m height slice, then samples were taken from the 49 m to 50 m slice, then 4 m to 5 m, then 48 m to 49 m etc. Acting on the assumption that the 3DBMs are fully or at least almost spatially complete, we further generated background samples of zero building height in order to familiarize the machine learning models with non-building surfaces, which are not included in the 3DBMs. To achieve this, we first generated a random point dataset across the 3DBM bounding box using Poisson Disc sampling (Tulleken, 2008). We subsequently eliminated all points that were closer than 100 m to any building part centroid in the full 3DBM.

#### 4.3.3 Modelling and validation

Both model training and the subsequent building height prediction were performed using FORCE (https://github.com/davidfrantz/force).

##### 4.3.3.1 Preliminary analysis

First, we inter-compared the performance of the radar features, the optical features, as well as a synergistic combination of both on the Berlin dataset. This dataset contains a large number of potential samples, as well as a good distribution among height classes (Table 2), which we attribute to Berlin being a diversely structured city with high-risers in the city center, very dense and compact neighborhoods in the inner city, and village-like settlements in the outskirts. We trained Support Vector Machine regression (SVR) models using 90% of the training data. The SVR hyper-parameters were tuned using grid search with 10-fold cross-validation. The remaining subsample was used for model inspection. A comparison of SVR with Random Forest regression can be found in the supplemental material section A1.

As the large feature space generated (1638 features) may result in overfitting and computational complexity related to the “curse of dimensionality” (Rust, 1997), we reduced dimensionality to 50 features using a two-step feature reduction approach as documented in supplemental material section A2.

##### 4.3.3.2 Final modelling

For producing the wall-to-wall building height map for Germany, we combined the training data from all available 3DBMs. First, we evaluated the model performance with a left-out 30% sample of the training dataset. As we did not have access to training data for complete Germany, we further explored the model's transferability and extrapolation capabilities by training models, where one training site was exclusively kept for model evaluation, whereas the remaining sites were used for training, respectively; this cross-validation approach yielded five models, i.e. each city was entirely left out once.

We generated density plots to visually compare the relationship between building heights and the reference, complemented by a number of statistical descriptors. Ideally, this relationship is linear and oriented along the one-to-one line of the density plot. We therefore fitted a linear function to the point cloud to statistically evaluate this relationship.

As the stratified sampling scheme was designed to collect the same number of validation samples for each meter of height, whenever possible, we used ordinary least squares (OLS) regression, which is a good estimator of how well the model is able to predict buildings of different height classes. The Root Mean Square Error (RMSE) based on this sample estimates the height class uncertainty. However, in relative terms, there are few high-rise buildings (only 6% of all Germany buildings are multi-family houses with more than 7 dwellings (Landesamt für Statistik Niedersachsen, 2014)). Therefore, the OLS estimate is skewed towards higher building heights. Consequently, we additionally computed weighted least squares (WLS) regressions wherein each building height class was weighted with the frequency of its occurrence in the corresponding 3DBM. The WLS estimate is more representative of the areal accuracy, e.g. when reporting a mean building height for a given area (e.g. a city, district or state). Accordingly, the weighted RMSE is a measure of the areal height uncertainty. Complementary to this, we computed both OLS and WLS regression through the origin, as building height is a parameter with a well-defined lower boundary.

We further compared the building height distribution for all 3DBMs using histograms, and investigated potential saturation effects with increasing height by computing and visualizing mean predicted building height per building height class in the reference datasets (using the cross-validation approach).

After assessing prediction quality, we trained the model on the full training sample (i.e. 100%) before model deployment for the wall-to-wall building height map production. The final building height prediction was further pruned at the lower end, i.e. all predictions <2 m were eliminated.

## 5 Results

### 5.1 Synergistic use of radar and optical data

**Fig. 6:**
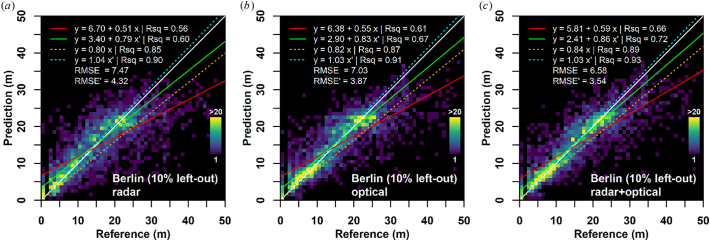
Support Vector Regression model comparison using radar-only (a), optical-only (b), and both data sources combined (c). White Line = one-to-one; red line: ordinary least squares regression, orange line: ordinary least squares regression through origin; green line: weighted least squares regression; cyan line: weighted least squares regression through origin; RMSE: Root Mean Squared Error, RMSE’ = weighted RMSE; weights were obtained from the frequency of occurrence within the reference dataset. (For interpretation of the references to colour in this figure legend, the reader is referred to the web version of this article.)

We trained SVR models based on all radar, optical, and combined radar and optical features, respectively. The radar-only model achieved an uncertainty (RMSE) of 7.47 m, and frequency-weighted uncertainty of 4.32 m, respectively (Fig. 6a). The optical-only model (b) performed slightly better in all statistics. A visual inspection of the density plot reveals that the density is better concentrated on the one-to-one line and the absence of buildings (no building, i.e. reference height = 0 m) is better predicted. The combination of radar and optical features (c) outperforms both single-domain models in every statistic, e.g., both RMSE measures have been reduced by nearly one meter when compared to the radar-only model. The density plot clearly reveals less scatter, an improved density concentration on the one-to-one line, as well as an increase in regression slope, which especially in the weighted cases is very close to the one-to-one line. A comparison of SVR with Random Forest regression can be found in the supplemental material section A1. Results for the feature selection are documented in the supplemental material section A2.

### 5.2 Building height validation and model transfer

**Fig. 7:**
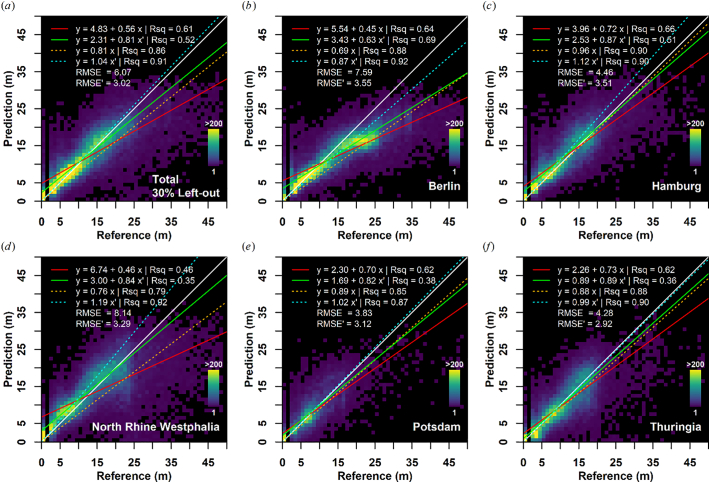
Building height validation on a left-out 30% sample of the global training dataset (a), on independent validation datasets left out for testing model extrapolation and transferability (b-f). White Line = one-to-one; red line: ordinary least squares regression, orange line: ordinary least squares regression through origin; green line: weighted least squares regression; cyan line: weighted least squares regression through origin; RMSE: Root Mean Squared Error, RMSE’ = weighted RMSE; weights were obtained from the frequency of occurrence within the reference dataset. (For interpretation of the references to colour in this figure legend, the reader is referred to the web version of this article.)

The performance of the global model (Fig. 7a) with a reduced feature set (trained and validated with a 70–30 data split with samples from all sites) is very similar to the local model comprised of the complete feature space (Fig. 6c). A model degradation due to feature reduction was not observed. On the contrary, most performance indicators point to an increase in prediction quality.

Fig. 7b-e illustrate the performance of the model when transferred to different areas and independent 3DBMs (not used for training). The height class uncertainty (RMSE) ranges from 3.83 m to 8.14 m, and the areal height uncertainty (frequency-weighted RMSE) is between 2.92 m and 3.55 m. In terms of OLS R^2^, the best correlation is found for the Hamburg dataset (R^2^ = 0.66). In other statistics, e.g. RMSE and RMSE’, the best performance is achieved in Potsdam or Thuringia.

**Fig. 8:**
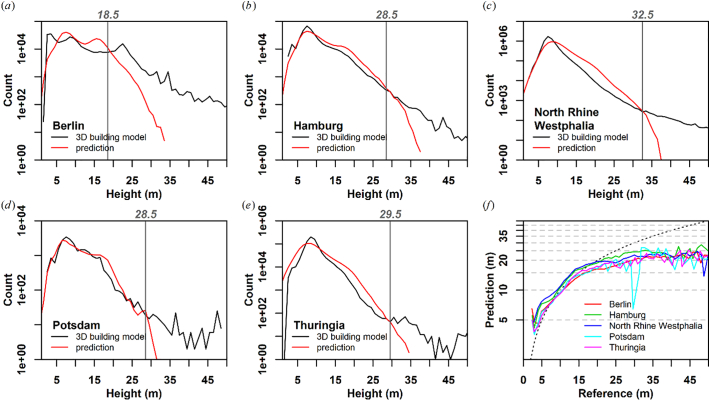
Histograms (a-e) and saturation effect (f). (a-e): the histograms are not based on the validation sample, but on the complete dataset (“Number of potential samples” line in Table 2), thus, they represent the complete statistical population. The y-axes are drawn logarithmic as the building height distributions are right-skewed, e.g. more than 96% of all buildings in Thuringia are lower than 15 m (Table 2). (f): mean predicted building height per building height class of the reference datasets (in 1 m increments). The y-axis is drawn logarithmic and the dashed curve represents the one-to-one line.

Histograms of the building height prediction and independent reference models (not used for training) are shown in Fig. 8a-e. For the city datasets, especially Hamburg and Potsdam, the height distribution is well matched. Almost all datasets are characterized by an overestimation of buildings above 10 or 15 m, which is most pronounced for the areal states of Thuringia and North Rhine Westphalia. Above a height of about 30 m however (vertical lines), the overestimation quickly turns into a substantial underestimation. This is also where the histogram becomes noisier due to decreasing sample sizes. In all datasets, a saturation effect is apparent at approximately 20 m reference height (Fig. 8f).

### 5.3 Building height mapping

**Fig. 9:**
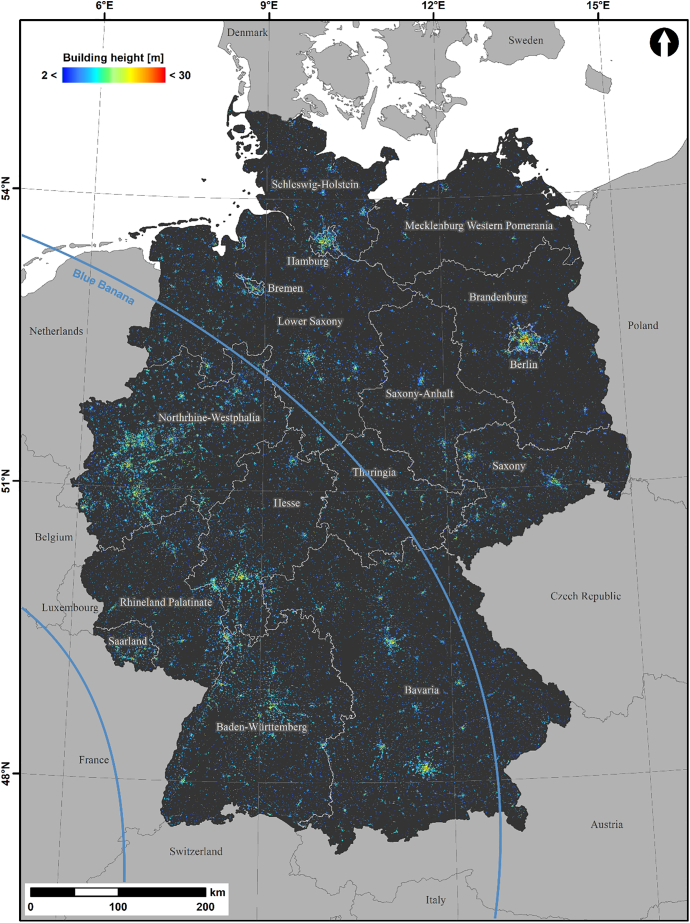
Building height map for Germany. The full dataset is openly available (Frantz et al., 2020), and can additionally be explored in the interactive map viewer at https://ows.geo.hu-berlin.de/webviewer/building-height/.

The wall-to-wall building height map for Germany is shown in Fig. 9. The full dataset is openly available (Frantz et al., 2020), and can additionally be explored in this interactive map viewer: https://ows.geo.hu-berlin.de/webviewer/building-height/. Large cities like Berlin, Hamburg or Munich are readily visible in the overview map, as are large urban agglomerations, e.g. along the blue banana (Brunet, 1989) that covers the Ruhr District, Frankfurt Rhine-Main, Rhine-Neckar, and Stuttgart metropolitan areas in Western Germany as indicated by the blue corridor. City centers are dominated by taller buildings, whereas city outskirts, smaller cities or the rural landscape are generally covered by smaller buildings.

**Fig. 10:**
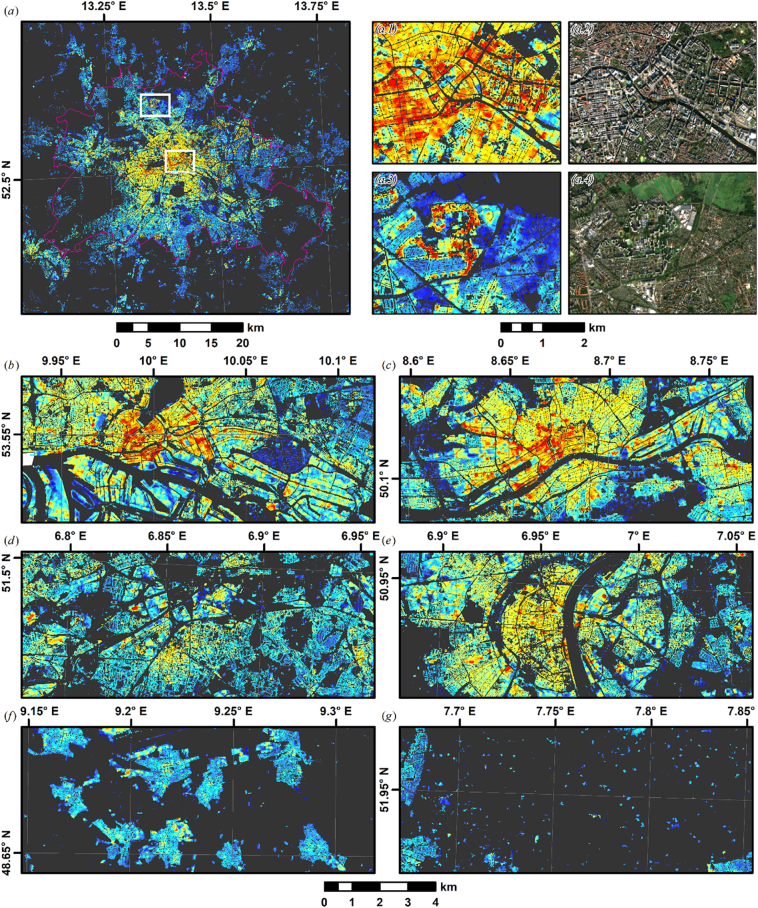
Building height close-ups. (a) Berlin with subsets depicting Berlin Center (a.1–2) and Berlin Reinickendorf (a.3–4) in the height map and in VHR true colour (Sentinel-2 Q50 STM); (b): Hamburg; (c): Frankfurt; (d): Ruhr District (Oberhausen); (e): Cologne; (f): nucleated settlements in Stuttgart metropolitan area; (g): dispersed settlements near Münster. The colour ramp corresponds to Fig. 9.

Fig. 10a is characterized by taller buildings in the city center and decreasing building heights towards the city limits. The close-up, e.g. Central Berlin (a.1) reveal that our approach resolves fine-scale structures like building blocks. Buildings along larger and higher-grade streets are generally taller. Berlin-Reinickendorf (Fig. 10a.3) is a district largely dominated by single-family houses. However, it also contains one of Berlin's largest housing estates, the “Märkisches Viertel”, where high-rise buildings clearly contrast with the surrounding single-family houses. Hamburg (Fig. 10b) is characterized by a contrast between the circular city core, the port (south of the river) and a large allotment to the East (dark blue patch). Frankfurt (Fig. 10c) is the only German city, where a skyline-dominating CBD is present (red area in the city core north of the river). On the contrary, many German cities, such as Cologne (Fig. 10d) are rather dominated by historic buildings, where building height is more uniform in the city center. While the Ruhr district (Fig. 10e, Germany's largest metropolitan area) is characterized by transitions between different cities, clearly distinct nucleated settlements in the Stuttgart metropolitan area are shown in Fig. 10f, although they are formally administered as single city (Filderstadt). Dispersed settlements in rural Münsterland (Fig. 10g) are dominated by separate farmsteads with rather low buildings scattered throughout the area.

### 5.4 Regional building height distribution

**Fig. 11:**
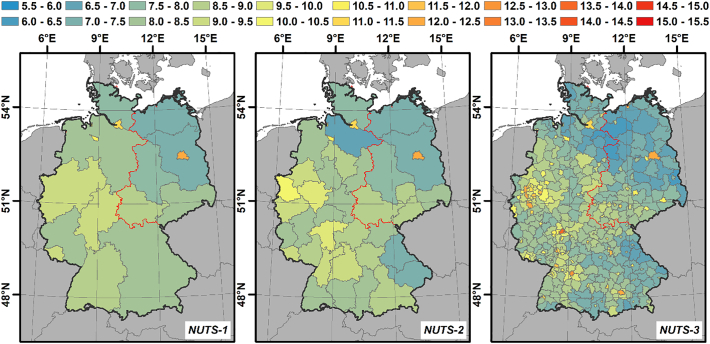
Mean building height per NUTS unit; NUTS-1 to 3 represent states, government regions and districts, respectively. The red line represents the former border between West and East Germany (the former Inner-Berlin border is not drawn for graphical reasons). (For interpretation of the references to colour in this figure legend, the reader is referred to the web version of this article.)

The mean building height for three NUTS (Nomenclature of Territorial Units for Statistics) levels is shown in Fig. 11. NUTS-1 to 3 represent European states, government regions and districts, respectively. On the NUTS-1 level, the city-states Berlin, Hamburg and Bremen show highest mean building height. The areal states with highest and lowest buildings are North Rhine Westphalia and Brandenburg, respectively. On the NUTS-3 level, districts with large cities, e.g. Frankfurt, Munich, Stuttgart, Cologne, Düsseldorf and several cities in the Ruhr district exhibit even higher building heights than the city states. Frankfurt hosts Germany's highest buildings in its banking district with a mean building height across the entire city district of 13.7 m. Areas along the blue banana belt are also covered with higher buildings than surrounding areas. In general, it appears that there is a trend towards lower buildings in East Germany, which may be a result of different planning policies before Germany's reunification. The more detailed NUTS levels indicate, however, that regions and districts with low building height are not exactly aligned with the former border between West and East Germany (red line). There are larger areas with low buildings in northern Lower Saxony and eastern Bavaria, too. In addition, building heights in southern Saxony and Thuringia do not differ substantially from other districts in West Germany.

## 6 Discussion

### 6.1 Synergistic use of radar and optical data

While radar is known to be sensitive to building height (Koppel et al., 2017), mapping from optical data was previously limited to VHR imagery, wherein either shadow measurement techniques or photogrammetric analysis was employed. However, as outlined in section 4.1, spatial and temporal patterns of building shadows are even observable in optical decameter resolution imagery. Our findings indicate that machine-learning based models are capable of predicting building height from both radar-only and optical-only data with similar performance, with a slightly better performance for optical data (Fig. 6). It appears that the optical-only results have less scatter in the density plot, and thus a smaller uncertainty (RMSE). In addition, the height in the absence of a building (reference height = 0 m) is better predicted in the optical case. Nevertheless, the synergistic combination of radar and optical data outperformed both single domain models.

It is known that the relationship between backscatter and building height is adversely affected by several effects. We aimed at reducing the effect of building orientation by using both polarizations (Li et al., 2020b), as well as by using data from both ascending and descending radar orbits (Koppel et al., 2017; Li et al., 2020a), which however may result in some uncertainty where viewing directions do not overlap (cf. Fig. 2b). Other geometric effects like urban layouts and structures causing double-bouncing effects, as well as overlay effects with increasing building density affect the results, too (Li et al., 2020b). Due to the side-looking view geometry, these effects cannot be fully compensated for when using radar data only, whereas such geometric effects are nearly non-existent in nadir-looking optical data. Unlike radar data, optical images are also not adversely affected by specific surface materials, e.g. specular reflectance over metal roofs that results in a loss of signal. On the contrary, the multi-spectral information likely provides explanatory power, as e.g. lower buildings in Germany are usually covered with roof tiles, whereas taller buildings often have flat roofs covered in other materials. Li et al. (2020b) further noted that tree canopies adversely affect building height predictions with radar data. We consider that optical imagery has both advantages and disadvantages with regards to trees. On the one hand, multi-spectral and multi-temporal observations are expected to provide explanatory power as e.g. suggested by Li et al. (2020a), as e.g. low single-family buildings are often embedded in settlement forms with a high share of tree cover. On the other hand, we approximate shadowing effects through texture metrics. Thus, any strong image contrast can potentially result in the prediction of spurious heights, e.g. along avenues. To minimize this adverse effect, our approach is best paired with existing settlement extent layers, which was also done by (Li et al., 2020a; Li et al., 2020b). In our case, we masked our final map with the European Settlement Map. Despite its challenges (some residential streets classified as building and a large missing data patch of 94.7 km^2^ in North Rhine Westphalia (51.497° N, 7.271° E), it is provided at optimal spatial resolution for our purpose, and is distributed with a free and open data license.

### 6.2 Prediction quality

#### 6.2.1 Validation of model

The quantitative validation on the left-out 30% global sample reveals a proportional relationship between predicted and reference height (Fig. 7a), which visually is close to the one-to-one line for a substantial part of the data range. The sampling scheme collected the same number of samples for each meter of height between 3 m and 50 m, wherever possible. Thus, OLS statistics between predicted and reference height are good estimators of prediction quality across different height classes. We observe a linear relationship (R^2^ = 0.61), thus our model is well capable of estimating building heights across most of the observed range of building height. However, the OLS estimates are skewed towards high-rise buildings, which in relative terms are rare in Germany. Thus WLS statistics, wherein each building height class was weighted with the frequency of its occurrence are more representative of the areal accuracy, and the corresponding RMSE is a measure of the areal height uncertainty. The WLS estimates are somewhat better than the OLS estimates: regression slope is closer to one, intercept closer to zero and RMSE is substantially lower (3.02 m vs 6.07 m), which all suggests good prediction quality for the most frequently occurring building heights.

#### 6.2.2 Model transfer (Transfer of model for national-scale mapping)

Validation on different areas with independent reference data (i.e. entirely left-out) suggests that the regional model transfer was successful and the resulting density plots (Fig. 7b-e) show a similar accuracy as the global validation (Fig. 7a). For some datasets, selected statistics are better, e.g. RMSE in Potsdam and Thuringia. Compared to all other datasets, both Potsdam and Thuringia have very few buildings taller than 20 m, which accordingly lowers RMSE, which is also reflected by smaller differences in the frequency-weighted RMSE between datasets.

The lowest transfer performance is observed for the state of North Rhine Westphalia with RMSE of 8.14 m, R^2^ of 0.46 and regression slope below 0.5. North Rhine Westphalia is the industrial core region of Germany; thus, many high-rising industrial buildings are located in this state, including tall factories but also many vertical structures like industrial smoke pipes, silos, or cooling towers. As we attempted to collect the same number of samples for each meter of height in the validation data, most of those tall infrastructures are included in the North Rhine Westphalia sample – although their relative proportion is comparably low (see Fig. 8). Accordingly, the OLS estimate is skewed to a larger degree as compared to the other datasets. This is readily visible in the density plots, wherein many buildings taller than 25 m are present, for which we additionally observe the most scatter (i.e. highest uncertainty). When applying the WLS-regression, the performance on the North Rhine Westphalia is much better, which suggests that the prediction of the most frequently occurring buildings is robust. The weighted RMSE is also more similar to the other datasets, which indicate that the areal height uncertainty is fairly uniform across Germany.

Our model shall be transferrable e.g. to neighboring countries that are characterized by similar building structures and material composition. From our results (especially the North Rhine Westphalia model transfer evaluation), we, however, expect that the model cannot be transferred without adaptations to regions that are too distinct from the settings in Germany, both in terms of climatic and structural properties (e.g. sky-scraper dominated cities in the U.S. or cities in deserts). When including such cases in the training sample (as e.g. by including samples from North Rhine Westphalia in the global model), we presume that the methodology might well be transferable to other parts of the globe. However, testing these hypotheses is out of scope of this paper, but merits future research.

#### 6.2.3 Saturation

Despite the proportional relationship between predicted and reference building height, we generally observe a saturation effect above 20 m (Fig. 8f). Note however, that these are average numbers, higher values than 20 m do exist in our results, and Fig. 8a-e) indicates that in terms of building height frequency on the city to state level, underestimation does not occur until about 30 m. This confirms findings from Koppel et al. (2017), who report a saturation at 20 m due to the overlay effect at 41–46° incidence angle in Sentinel-1 data on Tallinn. It appears that the additional optical data cannot fully compensate for this. This may be due to physical limitations, as e.g. shadows do not have a multiplicative darkening effect when a shadow overlays on other building's shadows. However, the saturation may simply be an effect of an insufficient number of high-rise buildings in our training data. Machine learning regression usually works best if the predicted values are well inside the trained range. We hypothesize that our method would account better for high-risers by significantly increasing their occurrence in the training dataset. This however, would need to be tested in other study areas (e.g. in the U.S.) as these building types are rare in Germany (cf. Table 2). For reporting statistics for larger areas, e.g. for administrative units, we consider this effect to be negligible due to the rare occurrence of this building category in Germany.

### 6.3 Inter-comparison with a recent 3D mapping product

Great advances have been made to map building height at spatial resolutions of 0.5 km to 1.0 km (Li et al., 2020a; Li et al., 2020b). Our maps presented in Fig. 9 and Fig. 10, as well as the online map (http://ows.geo.hu-berlin.de/webviewer/building-height) add to these advances by offering a further extension towards much higher spatial resolution. Our maps resolve fine scale built-up structures both in rural and urban contexts, e.g. different building blocks or even building footprints in the case of large buildings or farmsteads (see Fig. 10a.3 and g). However, it needs to be emphasized here that some care needs to be taken when interpreting this 10 m building height map. Our dependent variable is the average building height within a 50 m radius around the pixel of interest, thus, in dense and heterogeneous neighborhoods, we are still predicting a local mixture of different building heights. This is similar, although less pronounced, to previous approaches with coarser resolution. Our map can be readily aggregated to coarser spatial resolution to negate such issues if needed. Nevertheless, our approach allows for a comparative interpretation of highly detailed spatial patterns, which is not possible when directly mapping at coarse resolution.

**Fig. 12:**
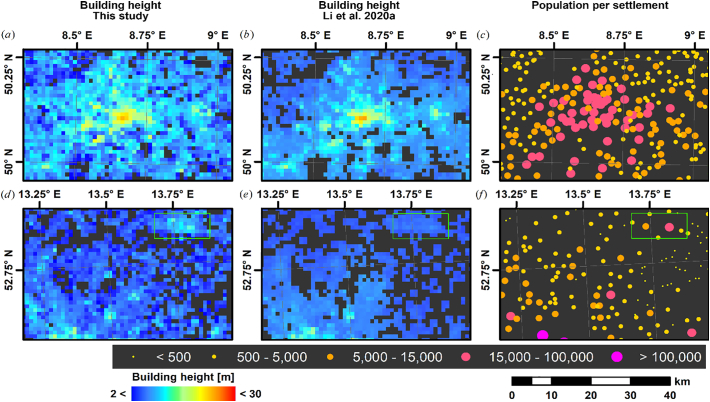
Inter-comparison of building height prediction with Li et al. (2020a) for Frankfurt Rhine-Main (a-c) and Berlin surroundings (d-f). (a,d): aggregated building height prediction as presented in this study; (b,e) building height prediction of Li et al. (2020a); (c,f) population per settlement (GeoBasis-DE / BKG, 2020).

We further inter-compared the final building height map with the 1 km map published by Li et al. (2020a), which was produced with a generalized, inter-continental model that has seen training data across the United States, China, and Europe. To homogenize the datasets as far as possible, we clipped the Li map to Germany, and aggregated our map to 1 km using average resampling (Fig. 12). Both approaches yield similar results for highly urbanized areas (see Fig. 12 top: Frankfurt Rhine-Main agglomeration). However, the predictions for rural and suburban areas are less congruent (see both Frankfurt Rhine-Main surroundings, as well as Berlin surroundings in the second row). Our map has fewer nodata values: this is a direct result of the different settlement masks and not related to the building height prediction. Still, outside of urban agglomerations, the Li map features very homogenous predictions, whereas our predictions are typically higher, and with more variability; see e.g. the differentiation between medium regional centers with ten thousands of residents (e.g. Eberswalde; green box) and small settlements.

**Fig. 13:**
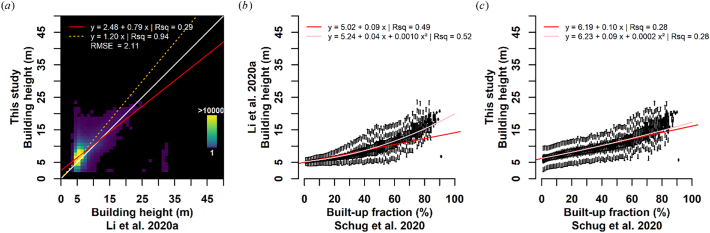
Inter-comparison of building height prediction with Li et al. (2020a). a): density plot; white Line = one-to-one; red line: ordinary least squares regression, orange line: ordinary least squares regression through origin; RMSE: Root Mean Squared Error; note the non-linear colour ramp. (b-c): boxplot of the building height predictions per built-up density (Schug et al., 2020), as well as statistical relationship between building height and built-up density. Only pixels with valid predictions in both datasets were investigated. (For interpretation of the references to colour in this figure legend, the reader is referred to the web version of this article.)

A portion of the data is stretched along the one-to-one line (Fig. 13); however, the vast majority of pixels are located in a cluster with ca. 5 m buildings in the Li dataset, for which our map indicates a larger variability. This results in a rather low statistical relationship with R^2^ = 0.27. Note that the disagreement at ca. 32 m in the Li map is a result of spurious estimates in surface mining areas, only affects a fairly small number of pixels, and is related to commission errors in both settlement masks. When stratifying building height predictions by the average built-up fraction within 1 km cells as derived from Schug et al. (2020), both predictions are fairly similar when built-up density is >50% (Fig. 13b-c); the linear relationship between the two predictions for these pixels yields R^2^ = 0.66, which is significantly higher than for the complete dataset (Fig. 13a). In the Li map, the variability of predicted building height decreases with decreasing built-up density, whereas the variability is fairly constant for all densities in our map. Also, the Li building height predictions are positively correlated with building density – as already reported by Li et al. (2020a). However, this relationship is much weaker in our case. We hypothesize that this difference is because of two main reasons:

• When predicting building height, uncertainty increases with resolution. This effect is well documented in Li et al. (2020b), and our findings quantify this uncertainty for our map (cf. Fig. 7a RMSE measures).
• There appears to be a loss of sensitivity for sparsely populated areas in the Li map. We hypothesize that this is because the “aggregate-then-predict” method hits a spectral-mixture related limit when buildings become underrepresented in a pixel. This seemingly results in a dependency of the building height prediction on building density. In our “predict-then-aggregate” case, the 1 km average may be composed of a single building, for which our approach is sensitive enough. However, due to bullet one, a larger uncertainty applies as compared to the other strategy.

In summary, both 1 km products are similar for highly urbanized areas, whereas our map shows more variation in rather sparsely populated areas due to the absent dependency on building density when originally mapping building height at high resolution.

### 6.4 Regional distribution

Across Germany, mean building height varies considerably (Fig. 11). Unsurprisingly, mean building height is higher for administrative units that are mostly comprised of large cities, e.g. Hamburg and Berlin on the state level, as well as other large cities like Frankfurt on the district level. The state level analysis implies that buildings are generally lower in Eastern Germany as compared to the West. The region and district analysis however suggests that this is unrelated to the former Inner-German border and thus not an effect of different socio-political systems. Instead, building height appears to be robustly dependent on population density (see Fig. A7 in the supplemental material), which is probably due to a mutual interdependence of economic pull and building height.

## 7 Conclusion

This study presented a methodology to predict building height using a synergistic combination of dual-polarized Sentinel-1A/B and multi-spectral Sentinel-2A/B time series using a 10 m grid resolving local structures both in rural and urban contexts. Our findings confirm our hypothesis that the combined usage of optical and radar data excels the usage of one data source alone. We employed machine learning regression to predict building height using highly accurate training and validation data derived from 3D building models. We rigorously reported on prediction quality as well as on the accuracy of spatially transferring the model. We have inter-compared our map with a recent dataset and found that differences are mostly due to differences w.r.t. the data aggregation strategy. We further showed that building height varies considerably across Germany with lower buildings in less densely populated areas in Eastern and South-Eastern Germany. As demonstrated in this paper, we emphasize the straightforward applicability of this approach on the national scale as it relies on freely available satellite imagery and open source software, which potentially permit frequent update cycles and cost-effective mapping that may be relevant for a plethora of different applications. We conclude that the application of our method could be especially beneficial in countries, where information on building height is only available for smaller areas.

## Declaration of Competing Interest

None.

## Appendix A Supplementary data

The dataset presented in this paper is openly available: https://zenodo.org/record/4066295#.X4QRNmgzYmI (Frantz et al., 2020). The data can additionally be explored in this interactive map viewer: https://ows.geo.hu-berlin.de/webviewer/building-height/. Image 1
