# Supplementary material for: National-scale mapping of building height using Sentinel-1 and Sentinel-2 time series
Source: Remote Sens Environ. 2021 Jan;252:112128. doi: 10.1016/j.rse.2020.112128 (PMC8190528; doi:10.1016/j.rse.2020.112128)
Supplement: Supplementary file 1 — The dataset presented in this paper is openly available: https://zenodo.org/record/4066295#.X4QRNmgzYmI (Frantz et al., 2020). The data can additionally be explored in this interactive map viewer: https://ows.geo.hu-berlin.de/webviewer/building-height/. [file mmc1.pdf]

Remote Sensing of Environment

Supplemental Material

National-scale mapping of building height using Sentinel-1+2 time series

David Frantz[a*], Franz Schug[a,b], Akpona Okujeni[a], Claudio Navacchi[c], Wolfgang Wagner[c], Sebastian van der Linden[d], Patrick Hostert[a,b]

[a] Earth Observation Lab, Geography Department, Humboldt-Universität zu Berlin, Unter den Linden 6, 10099 Berlin, Germany.

[b] Integrated Research Institute on Transformations of Human Environment Systems (IRI THESys), Humboldt-Universität zu Berlin, Unter den Linden 6, 10099 Berlin, Germany.

[c] Department of Geodesy and Geoinformation, TU Wien, Wiedner Hauptstraße 8/E120, 1040 Vienna, Austria

[d] Institute of Geography and Geology, University of Greifswald, Friedrich-Ludwig-Jahn-Str. 16, 17489 Greifswald, Germany

[*] Corresponding author. *E-mail address:* david.frantz@geo.hu-berlin.de (D. Frantz).

Structure

A1: Choice of machine learning method

A2: Feature selection

A3: Mean building height and population density

A1. Choice of machine learning method

Random Forest Regression (RFR) and Support Vector Regression (SVR) are two widely used machine learning methods employed in the field of remote sensing. Based on the Berlin dataset, we have evaluated which method to use for predicting building height. This assessment complements the preliminary analysis as presented in section 4.3.3.a. and 5.1 of the main paper. We inter-compared the performance of the radar features, the optical features, as well as a synergistic combination of both on the Berlin dataset. For this, we tested two machine learning methods:

1) We trained SVR models using 90% of the training data. The SVR hyper-parameters were tuned using grid search with 10-fold cross-validation. The remaining subsample was used for model inspection. For clarity of presentation, Figure 6 from the main paper is reproduced here as *Figure A*1.
2) Analogous, we trained RFR models with 500 trees of maximal depth, and used a third of all available features at each tree node to find the optimal splits (Figure A2).

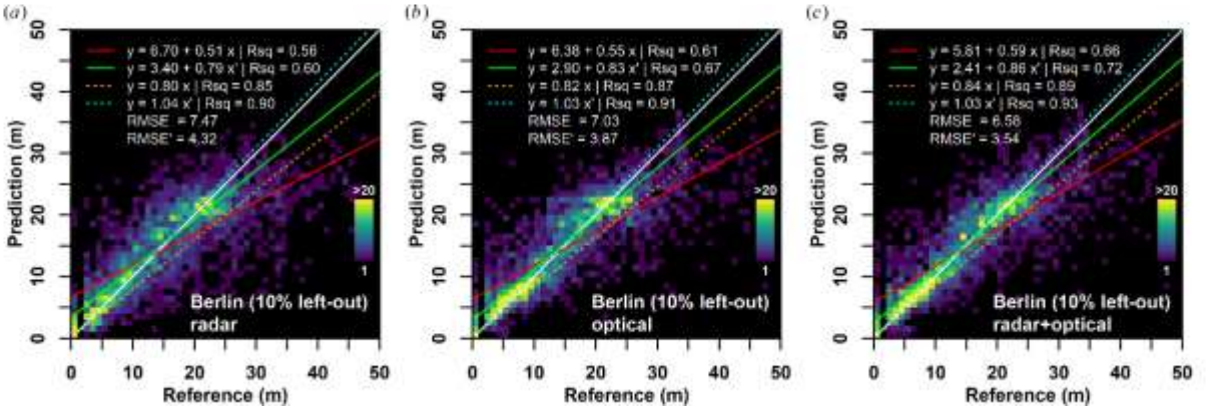

Figure A1. Support Vector Regression model comparison using radar-only (a), optical-only (b), and both data sources combined (c). White Line = one-to-one; red line: ordinary least squares regression, orange line: ordinary least squares regression through origin; green line: weighted least squares regression; cyan line: weighted least squares regression through origin; RMSE: Root Mean Squared Error, RMSE' = weighted RMSE; weights were obtained from the frequency of occurrence within the reference dataset

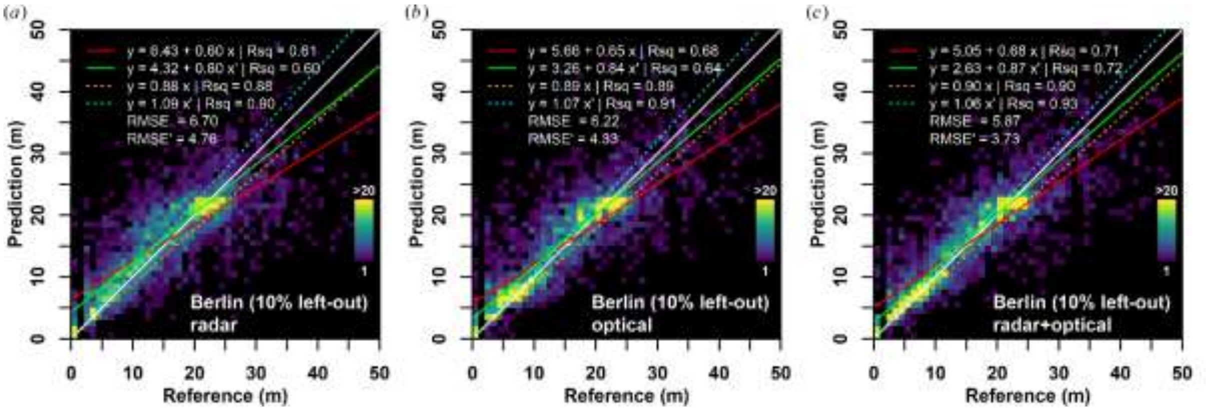

Figure A2. The same as Figure A1, but using Random Forest Regression.

The OLS estimates suggest the superiority of RFR as compared to SVR. However, the frequency-weighted RMSE indicates the opposite. When inspecting the map representation of the building height prediction, we observed a largely consistent behavior between the methods, except for large commercial buildings (*Figure A*3). We presume that this is because the buildings are larger than the radius used for generating the morphological metrics, and as such, the prediction can only rely on the spectral/backscatter signal of the roofing material (i.e. no building shadows nor trihedral scattering mechanisms are present). Obviously, the RFR substantially overestimated building height in these cases, whereas more reasonable heights were predicted by SVR – although not highly accurate either.

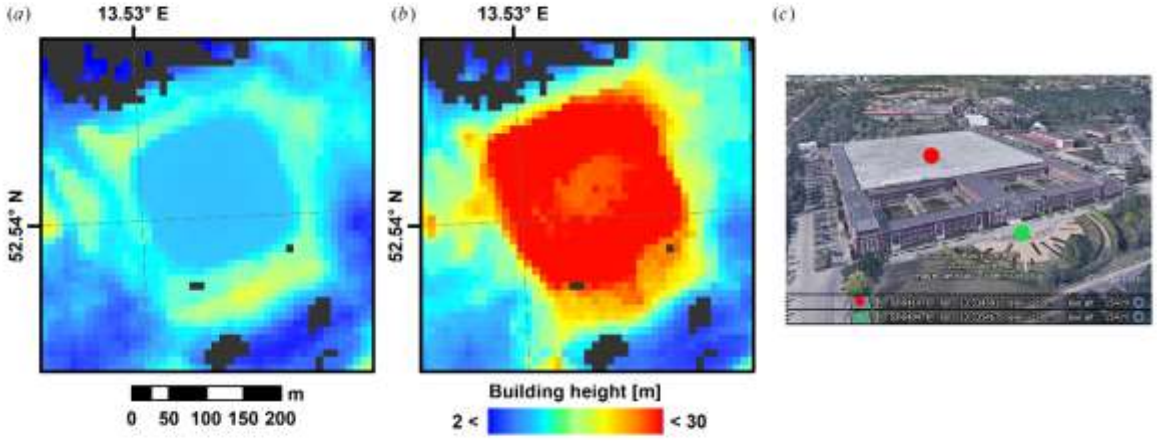

Figure A3. Building height prediction using the combined radar + optical model using Support Vector Regression (a) and Random Forest Regression (b) for a large commercial building in Berlin as shown in c): © Google Earth.

This is further corroborated by *Figure A*4. The mean absolute percentage error indicates that RFR is performing worse for specific building heights, e.g. for short buildings (4-5 m) as well as for 9-12 m tall buildings, which appears to be a typical height for commercial buildings. For tall buildings > 30 m however, RFR may be superior. However, due to the rare occurrence of these buildings in Germany, we opted to select SVR for producing the wall-to-wall building height map.

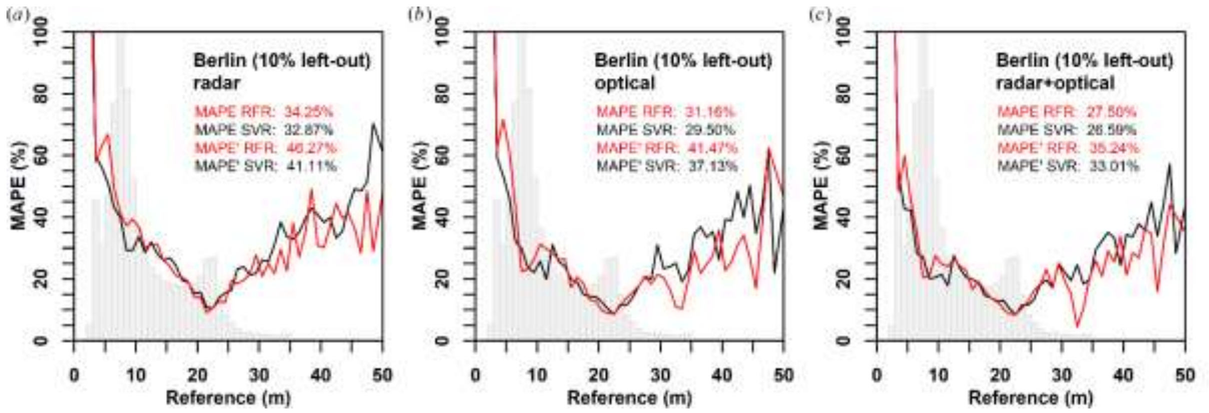

Figure A4. Mean Absolute Percentage Error (MAPE) per reference height for the radar-only (a), optical-only (b), and combined models (c). Black Line = Support Vector Regression (SVR); red line: Random Forest Regression (RFR); grey bars: histogram of reference building height. The legend displays the total MAPE, as well as the MAPE weighted by the frequency of occurrence within the reference dataset.

A2. Feature selection

1) Methods

a) Pre-selection

The large feature space generated may result in overfitting and computational complexity related to the "curse of dimensionality" (Rust 1997). As a consequence, we reduced dimensionality using a two-step feature reduction approach. In the first step, we aimed at quickly eliminating the least important features using a fairly fast training technique that provides a feature ranking. For this, we employed a Random Forest regression (RFR) on a random 10% subsample (500 trees, 1% of features tested at each split for fast computation) and retained all features that contribute more than 1‰ feature importance, i.e. we only removed features that did not contribute information at all.

b) Model selection

In the second step, we switched to our target machine learning algorithm, i.e. Support Vector Machine regression (SVR). Similarly to Schug et al. (2020), feature reduction was achieved by repeatedly training models using random feature subsets. We trained 100 SVR models, and randomly selected 50 pre-selected features as a reasonable compromise between testing many feature combinations and performing costly feature reduction strategies like forward, backward, or exhaustive feature selection. For each mode, the SVR hyper-parameters were tuned using grid search with 10-fold cross-validation. The training was performed on 90% of the training data.

The remaining subsample was used for model selection, wherein the model with best performance in terms of regression slope, offset, R^2, and RMSE was eventually selected.

2) Results

a) Pre-selection

Figure A5a-c shows the feature importance for the three different feature dimensions, i.e. spectral (a), temporal (b), and spatial (c) domains. Within each domain, boxplots are grouped according to spectral band, statistical aggregation and morphological metric, respectively. Within each domain, there are features that contribute more than others. Highest spectral contribution stems from the SAR data wherein both polarizations show high feature importance. However, optical data contribute valuable information, too. Especially vegetation-sensitive bands/indices contribute more, e.g. the near infrared band, NDVI or Tasseled Cap Greenness. Other optical indices seem to contribute less, which are mainly shortwave infrared bands, the first red-edge band, and Tasseled Cap Wetness. In the temporal domain, all metrics that indicate temporal snapshots and average state contribute equally well. The variability and distribution parameters contribute less. In the spatial domain, clear patterns are present. Blackhat and tophat metrics are least important, while closing, erosion and opening metrics contribute most information to the model. Within each group, positive outliers are present, which indicate a high usefulness of specific domain combinations. Thus, when selecting all features that contribute more than 1‰ feature importance, almost no group is entirely eliminated. However, more important groups are selected at a higher rate (Figure A5d-f), e.g. SAR bands, temporal snapshot statistics or erosion and dilation metrics. The full list of pre-selected features with feature importance ranking is presented in Table A1.

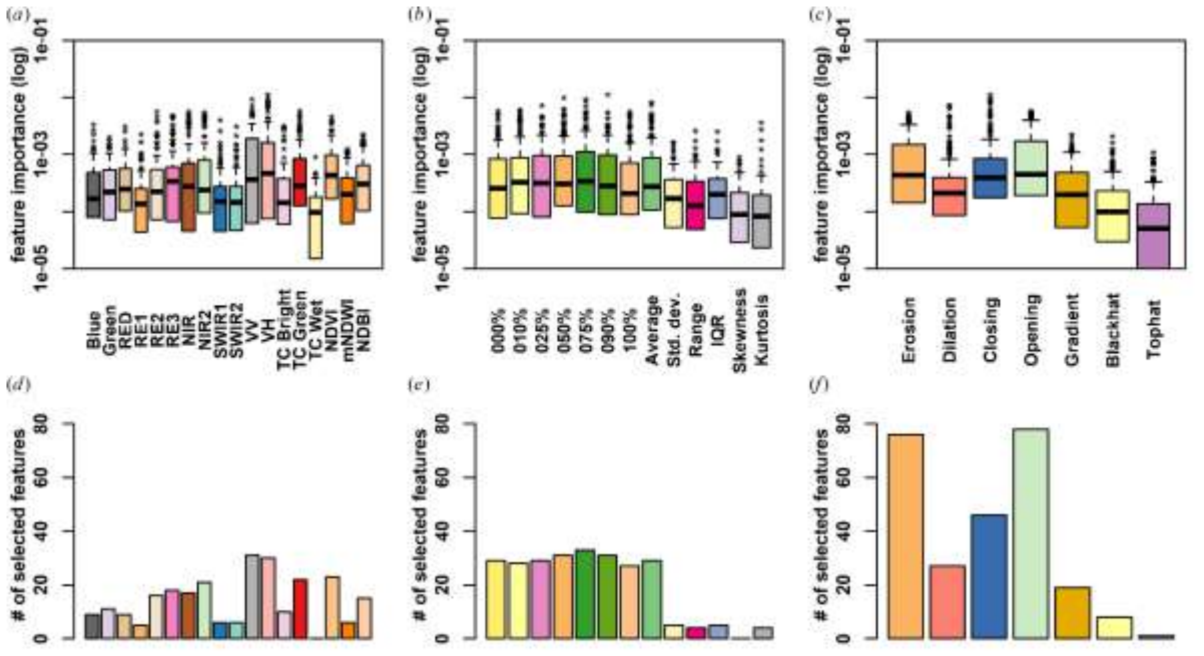

Figure A5. Feature pre-selection using Random Forest Feature Importance. Top: Boxplots of Feature Importance for the spectral (a), temporal (b) and spatial (c) domains, represented through bands/indices, aggregation statistics, and texture metrics, respectively; the y-axis is drawn logarithmic, thus outliers with 0% Feature Importance are omitted from this plot. Bottom: Number of selected features for the spectral (d), temporal (e) and spatial (f) domains. See Table A1 for a list with all pre-selected features.

b) *Model selection*

The performance indicators vary considerably from model to model (Figure A6a-d), indicating that not every sub-combination of features is equally meaningful. Most reduced models perform worse than the full model using all 1,638 features (horizontal red lines). However, a few feature subsets do indeed perform better, indicating that some features interact adversely. The selection of the best model is trivial as it showed superior performance in all chosen indicators (bullseye signature).

The 50 randomly selected features of the best performing model are shown in Figure A6, grouped according to domain (see supplemental material for the full list). Several groups were entirely eliminated, while other groups are selected at higher or lower rates than present in the pre-selection. Among high-ranking groups in the pre-selection, NDVI was entirely eliminated and median and maximum statistics were selected at a lower rate. Low-wavelength bands (blue, green), the 2nd red-edge band, kurtosis and closing were selected at a higher rate as compared to the pre-selection. The full list of selected features is presented in Table A2

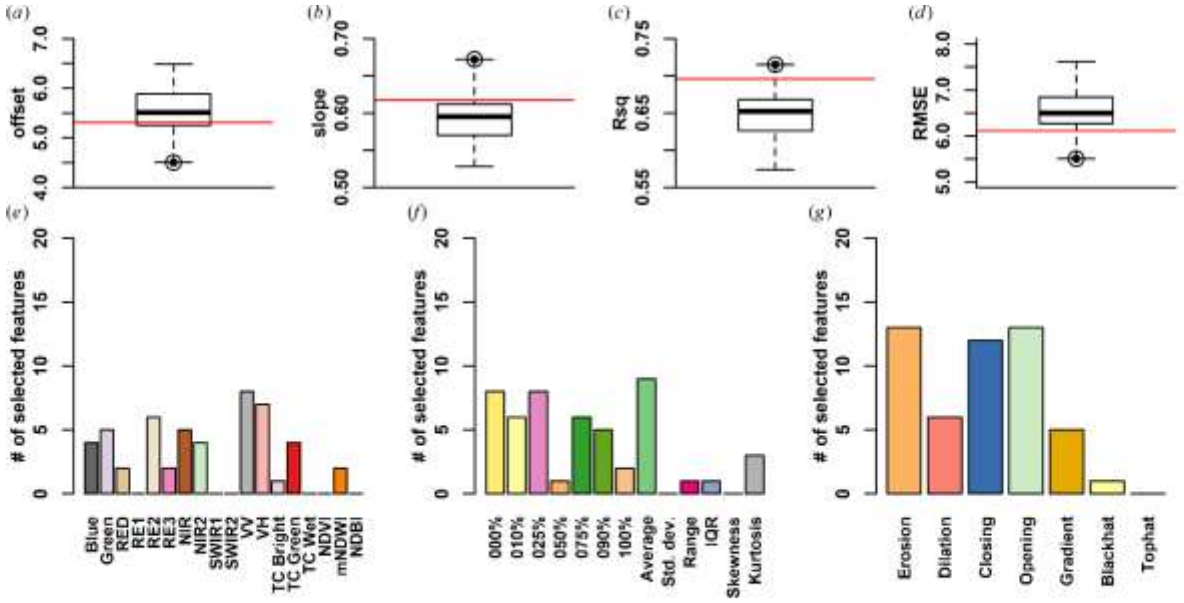

Figure A6. Model selection using Support Vector Machine Regression. Top: Model performance of 100 models trained on 50 randomly chosen feature subsets in terms of regression offset (a), slope (b), R^2 (c) and RMSE (d); the best-performing model is highlighted with bull's eye signature; the full model as shown in Figure 6f is highlighted with the red horizontal line. Bottom: feature subset of the best-performing model; number of selected features for the spectral (e), temporal (f) and spatial (g) domains. See Table A2 for a list with all 50 selected features.

3) *Discussion*

Along all feature dimensions, i.e. spectral, temporal and spatial domains, distinct features were selected at a higher rate. In the spectral dimension, both radar polarizations appear very relevant. This is likely due to the fairly direct relationship between backscatter and vertical structure (Li et al. 2020) There are also important optical bands and indices. Low-wavelength bands were likely selected as they capture brightness gradients, and vegetation-sensitive bands and indices were selected. These bands likely provide explanatory power to the machine learning model with regards to typical roof materials and vegetation compositions of particular settlement types. Short-wave infrared bands were not selected at all, which probability is due to its sensitivity to vegetation and water content, which is already covered by the near-infrared bands or not relevant for predicting building height, respectively.

Within the temporal dimension, temporal variability and data distribution statistics were selected at a lower rate, whereas most quantiles and the average are very important. The quantiles represent different temporal snapshots, and their combination implicitly include the temporal variability while also providing spectral information. Variability and distribution statistics were likely not selected as they provide redundant information

when several quantiles are available. Similarly, the median was hardly selected, whereas the average was very dominant, which is likely due to redundancy.

In the spatial dimension, two texture metrics were hardly selected (blackhat, tophat), while three metrics appear very important (erosion, opening, closing). The tophat and blackhat metrics are operations that enhance bright and dark elements in a dark and bright background, respectively. When applied to highly structured urban landscapes, the resulting images appear sharpened, which is probably an undesired property as most information content is rather encoded in the spatial neighborhood (e.g. shadow effects). The erosion (dilation) operation assigns the minimum value in the structuring element to the central pixel, i.e. in a city, it selects the darkest pixel / shadow (brightest pixel / roof). The opening operator performs a dilation on the eroded image, i.e. in a city, it selects the brightest of the selected shadows, whereas the closing operator preforms an erosion on the dilated image, i.e. in a city, it selects the darkest of the selected roofs. By combining those three texture metrics with each other, one can assume that some logical height derivation is possible, especially if complementary spectral and temporal information is added.

4) Full list of selected features

a) Pre-selection

Table A1. Pre-selected features using Random Forest feature importance ranking.

Rank	Band/Index	Aggregation Statistic	Texture Metric	Feature Importance
1	VH	90%	Closing	0.011183
2	VH	50%	Closing	0.009906
3	VV	75%	Closing	0.009331
4	VH	75%	Closing	0.008569
5	VH	Average	Closing	0.008182
6	VH	Average	Dilation	0.007332
7	VV	25%	Closing	0.007228
8	VV	25%	Dilation	0.007205

9	VH	75%	Dilation	0.006987
10	VH	50%	Dilation	0.006302
11	VV	10%	Closing	0.006048
12	VV	50%	Closing	0.006022
13	TC Green	0%	Opening	0.005762
14	NIR	75%	Opening	0.005686
15	RE2	10%	Opening	0.005669
16	VH	100%	Closing	0.005659
17	VV	90%	Opening	0.005446
18	NIR2	75%	Opening	0.005398
19	NIR2	90%	Erosion	0.005383
20	NIR2	Average	Erosion	0.005233
21	VV	100%	Opening	0.005159
22	TC Green	50%	Opening	0.00506
23	VV	100%	Closing	0.005044
24	VV	0%	Closing	0.004993
25	NIR	Average	Opening	0.004884
26	RE3	75%	Opening	0.004871
27	NIR2	10%	Opening	0.004866
28	NIR2	50%	Erosion	0.004842
29	VV	Average	Closing	0.004751
30	VV	75%	Dilation	0.004743
31	NIR	100%	Opening	0.004738
32	TC Green	25%	Opening	0.004705
33	VV	Average	Opening	0.004696

34	VV	10%	Dilation	0.004659
35	NIR2	50%	Opening	0.004634
36	RE3	50%	Erosion	0.004629
37	NDVI	Average	Opening	0.004592
38	RE2	0%	Opening	0.004566
39	VH	25%	Closing	0.004427
40	NIR2	90%	Opening	0.004343
41	NIR	25%	Opening	0.004325
42	NIR2	100%	Erosion	0.00432
43	RE3	Average	Opening	0.004276
44	VH	25%	Dilation	0.004274
45	RE3	90%	Opening	0.004273
46	NIR2	75%	Erosion	0.00426
47	TC Green	10%	Opening	0.004218
48	SWIR1	75%	Erosion	0.004001
49	NIR2	10%	Erosion	0.003994
50	NDVI	0%	Opening	0.00395
51	NIR	Average	Erosion	0.003941
52	NIR	10%	Opening	0.003913
53	RE2	50%	Erosion	0.003815
54	RE2	Average	Erosion	0.003752
55	NIR2	Average	Opening	0.00375
56	TC Green	Average	Opening	0.003738
57	NIR	100%	Erosion	0.003737
58	NDVI	10%	Opening	0.003725

59	VH	10%	Dilation	0.003677
60	NIR	50%	Erosion	0.00362
61	VH	Kurtosis	Opening	0.003618
62	VH	10%	Closing	0.003559
63	NIR	50%	Opening	0.00349
64	VV	25%	Opening	0.003451
65	NDVI	50%	Opening	0.003375
66	VH	90%	Dilation	0.003367
67	Blue	Average	Closing	0.00334
68	RE3	90%	Erosion	0.003323
69	VH	75%	Opening	0.003277
70	NIR	25%	Erosion	0.003271
71	NIR2	25%	Opening	0.003266
72	NIR2	100%	Opening	0.003217
73	TC Green	75%	Erosion	0.003214
74	NIR	90%	Opening	0.003184
75	TC Bright	100%	Erosion	0.003146
76	VV	75%	Opening	0.003117
77	VV	0%	Dilation	0.0031
78	VV	90%	Closing	0.003094
79	TC Bright	50%	Erosion	0.003082
80	SWIR2	25%	Erosion	0.003072
81	RED	10%	Closing	0.003054
82	TC Green	100%	Opening	0.003034
83	NIR2	0%	Opening	0.003018

84	RE3	100%	Erosion	0.002983
85	RE3	Average	Erosion	0.00297
86	VH	100%	Opening	0.002951
87	TC Bright	75%	Erosion	0.002849
88	TC Bright	Average	Erosion	0.002829
89	VV	90%	Dilation	0.002825
90	VV	Average	Dilation	0.002805
91	TC Bright	90%	Erosion	0.002805
92	NIR	75%	Erosion	0.002803
93	TC Green	10%	Closing	0.002775
94	VV	100%	Erosion	0.002774
95	VH	Kurtosis	Erosion	0.002753
96	TC Bright	50%	Opening	0.002715
97	NDVI	0%	Erosion	0.002715
98	TC Green	50%	Erosion	0.002705
99	Blue	10%	Closing	0.002692
100	TC Green	75%	Opening	0.002627
101	TC Green	90%	Opening	0.002605
102	NDVI	Range	Closing	0.002578
103	RE3	25%	Erosion	0.00257
104	SWIR1	50%	Erosion	0.002567
105	VH	IQR	Closing	0.002548
106	RED	0%	Closing	0.002528
107	RE3	75%	Erosion	0.002522
108	NIR	0%	Opening	0.00251

109	TC Green	10%	Erosion	0.002454
110	VV	50%	Opening	0.002424
111	RE2	90%	Erosion	0.002421
112	RE3	25%	Opening	0.002351
113	NDBI	50%	Closing	0.002279
114	VH	0%	Closing	0.002277
115	RE3	100%	Opening	0.002263
116	RE1	Average	Erosion	0.00223
117	Blue	0%	Gradient	0.002208
118	RE3	10%	Erosion	0.002202
119	RE2	75%	Opening	0.002196
120	RE2	50%	Opening	0.002182
121	NDVI	25%	Opening	0.002177
122	RE2	90%	Opening	0.002171
123	SWIR2	50%	Erosion	0.002134
124	NIR	0%	Erosion	0.002134
125	RE2	Average	Opening	0.002133
126	TC Bright	75%	Opening	0.00213
127	NIR2	100%	Blackhat	0.002094
128	VH	Average	Opening	0.002058
129	VV	50%	Dilation	0.002058
130	TC Green	25%	Erosion	0.002051
131	RE2	100%	Erosion	0.00205
132	RE2	10%	Erosion	0.002043
133	NIR2	25%	Erosion	0.002039

134	NDBI	100%	Closing	0.002032
135	VV	Average	Erosion	0.001995
136	RE2	75%	Erosion	0.00199
137	Green	0%	Gradient	0.00199
138	NDVI	75%	Opening	0.001954
139	NDVI	90%	Erosion	0.001858
140	NDBI	10%	Closing	0.001834
141	RE3	0%	Opening	0.001809
142	TC Green	Average	Erosion	0.001805
143	VH	100%	Erosion	0.001805
144	VV	Kurtosis	Opening	0.001775
145	VV	10%	Opening	0.001774
146	TC Bright	25%	Erosion	0.001773
147	RED	75%	Closing	0.001768
148	Green	75%	Erosion	0.001748
149	VH	50%	Opening	0.001738
150	TC Bright	100%	Opening	0.001733
151	NDBI	Range	Closing	0.001717
152	Blue	0%	Dilation	0.001706
153	RE3	IQR	Opening	0.001702
154	NDBI	75%	Closing	0.001699
155	TC Green	0%	Closing	0.001673
156	RE1	50%	Erosion	0.001658
157	RE1	75%	Erosion	0.001653
158	NDBI	50%	Dilation	0.001642

159	VH	Std. dev.	Gradient	0.001642
160	NDVI	100%	Erosion	0.001639
161	TC Green	0%	Erosion	0.001638
162	NDBI	90%	Closing	0.001637
163	NDBI	Average	Closing	0.001634
164	NIR2	Std. dev.	Blackhat	0.001612
165	RE2	100%	Opening	0.001604
166	SWIR1	25%	Erosion	0.001595
167	VH	100%	Dilation	0.001584
168	VV	90%	Erosion	0.001571
169	NDBI	90%	Opening	0.001547
170	RE2	25%	Erosion	0.001541
171	TC Green	75%	Closing	0.001535
172	NDBI	75%	Opening	0.001532
173	NIR	90%	Erosion	0.001522
174	RE3	50%	Opening	0.001518
175	NDBI	0%	Closing	0.001503
176	SWIR2	10%	Erosion	0.001501
177	NDVI	50%	Erosion	0.001499
178	NDVI	100%	Opening	0.001496
179	Green	Average	Erosion	0.00149
180	NDVI	10%	Closing	0.001472
181	NDVI	90%	Opening	0.001469
182	NIR	10%	Erosion	0.001468
183	RED	0%	Gradient	0.001455

184	RE3	10%	Opening	0.001454
185	SWIR2	100%	Erosion	0.001453
186	NDBI	Average	Dilation	0.001442
187	Green	10%	Dilation	0.001433
188	NIR2	90%	Gradient	0.001425
189	NDVI	25%	Erosion	0.001413
190	Green	10%	Gradient	0.001407
191	VV	75%	Erosion	0.001406
192	NDVI	90%	Closing	0.001387
193	Blue	25%	Closing	0.00138
194	NIR	Std. dev.	Dilation	0.001368
195	SWIR1	90%	Erosion	0.001363
196	VH	90%	Opening	0.001358
197	TC Green	100%	Erosion	0.001341
198	NDVI	Average	Erosion	0.001334
199	RE2	Range	Opening	0.001298
200	Green	25%	Dilation	0.001296
201	RE2	25%	Opening	0.001275
202	Blue	10%	Dilation	0.001273
203	VV	25%	Erosion	0.001255
204	Green	50%	Erosion	0.001253
205	NDBI	0%	Dilation	0.001252
206	TC Green	75%	Blackhat	0.001251
207	RED	50%	Gradient	0.001248
208	mNDWI	Average	Gradient	0.001242

209	TC Bright	90%	Opening	0.00124
210	VH	0%	Dilation	0.001234
211	NDVI	IQR	Blackhat	0.001226
212	VV	Kurtosis	Erosion	0.001223
213	SWIR2	25%	Closing	0.001218
214	SWIR1	100%	Opening	0.001215
215	SWIR1	Average	Opening	0.001192
216	NDVI	Std. dev.	Closing	0.001191
217	NDBI	90%	Dilation	0.001178
218	Blue	10%	Gradient	0.001178
219	RED	10%	Gradient	0.00117
220	mNDWI	75%	Gradient	0.001163
221	SWIR2	50%	Opening	0.001161
222	RED	75%	Erosion	0.001157
223	VH	75%	Gradient	0.001156
224	mNDWI	50%	Gradient	0.001154
225	RE3	0%	Erosion	0.001147
226	VH	IQR	Dilation	0.00114
227	NDVI	90%	Gradient	0.001139
228	NDBI	100%	Opening	0.001136
229	VV	75%	Gradient	0.001127
230	RED	25%	Gradient	0.001127
231	TC Green	25%	Closing	0.001121
232	NIR2	IQR	Blackhat	0.001119
233	mNDWI	25%	Gradient	0.001106

234	VV	0%	Opening	0.001101
235	VH	50%	Tophat	0.001088
236	RED	90%	Erosion	0.001084
237	TC Green	50%	Closing	0.001081
238	NDVI	75%	Erosion	0.001076
239	mNDWI	0%	Gradient	0.001072
240	RE3	Std. dev.	Blackhat	0.001065
241	Blue	25%	Dilation	0.001055
242	Green	10%	Closing	0.001055
243	RE1	100%	Erosion	0.001055
244	NDVI	0%	Closing	0.001044
245	Blue	Average	Gradient	0.001043
246	NIR2	0%	Erosion	0.00104
247	VH	0%	Opening	0.001038
248	Green	25%	Closing	0.001022
249	Green	0%	Closing	0.001012
250	mNDWI	Range	Closing	0.001012
251	Green	Average	Opening	0.001012
252	NDVI	0%	Dilation	0.001009
253	RE1	90%	Opening	0.001005
254	NIR2	90%	Blackhat	0.001004
255	TC Green	90%	Blackhat	0.001004

b) Model selection

Table A2. Selected features using evaluation of random feature subsets. Note that this method does not provide a feature ranking, i.e. the order of features in this list is arbitrary.

**Band/Index**	**Aggregation Statistic**	**Texture Metric**
VH	Average	Opening
RED	90%	Erosion
NIR2	100%	Blackhat
TC Green	50%	Closing
VV	10%	Opening
RE3	75%	Erosion
NIR2	90%	Erosion
VV	Average	Closing
Blue	0%	Gradient
Green	0%	Closing
TC Green	Average	Opening
TC Green	0%	Closing
RE2	75%	Erosion
RE2	90%	Opening
Blue	25%	Closing
TC Bright	25%	Erosion
NIR	Average	Opening
RE3	Average	Erosion
Green	Average	Erosion
RE2	10%	Opening
NIR	10%	Opening
VV	75%	Closing
VV	75%	Closing

mNDWI	25%	Gradient
VV	Kurtosis	Opening
VH	Average	Closing
VH	25%	Dilation
Blue	0%	Dilation
NIR	90%	Opening
Blue	25%	Dilation
TC Green	25%	Erosion
NIR2	75%	Erosion
RE2	10%	Erosion
VH	Average	Dilation
VV	Kurtosis	Opening
VH	10%	Closing
VV	0%	Dilation
NIR	0%	Erosion
NIR2	90%	Gradient
RE2	Range	Opening
VH	IQR	Dilation
VV	Kurtosis	Opening
mNDWI	25%	Gradient
NIR	10%	Erosion
Green	0%	Closing
Green	Average	Opening
VH	25%	Closing
RE2	100%	Erosion

RED	75%	Closing
Green	0%	Gradient

A3. Mean building height and population density

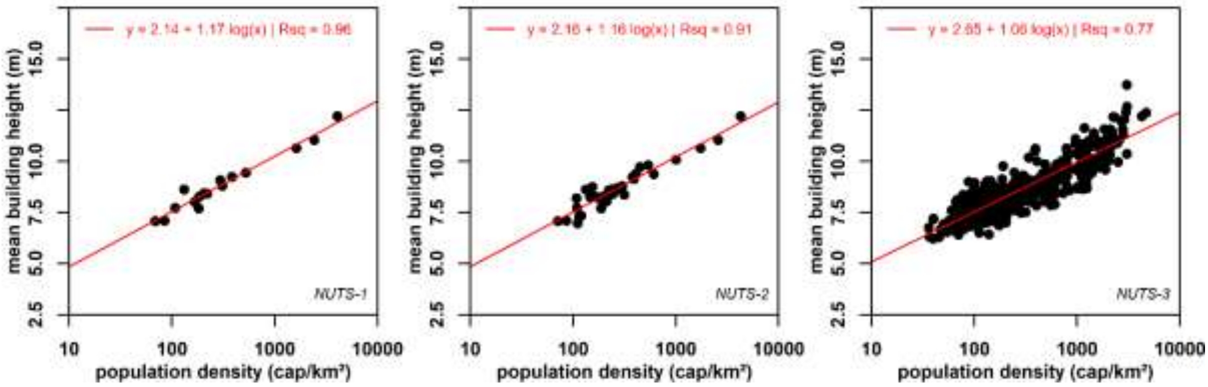

Figure A7. Population density vs. mean building height in NUTS units. NUTS-1 to 3 represent states, government regions and districts, respectively. x-axes are drawn logarithmic. Population density was acquired from official German and European statistical surveys (Eurostat 2020a, b; Statistische Ämter des Bundes und der Länder 2020). **[please insert horizontally as 2-column figure]**

References

1 Eurostat. (2020a). *Population density by NUTS 2 region*. Available: http://data.europa.eu/88u/dataset/QEgn3fJF0SQo7qpAN8T9g

2 Eurostat. (2020b). *Population density by NUTS 3 region*. Available: http://data.europa.eu/88u/dataset/GnGfvPQmfu5n6aKVXQkPw

3 Li, X., Zhou, Y., Gong, P., Seto, K.C., & Clinton, N. (2020). Developing a method to estimate building height from Sentinel-1 data. *Remote Sensing of Environment, 240*, 111705. DOI: 10.1016/j.rse.2020.111705

4 Rust, J. (1997). Using Randomization to Break the Curse of Dimensionality. *Econometrica, 65*, 487-516. DOI: 10.2307/2171751

5 Schug, F., Frantz, D., Okujeni, A., van der Linden, S., & Hostert, P. (2020). Mapping urban-rural gradients of settlements and vegetation at national scale using Sentinel-2 spectral-temporal metrics and regression-based unmixing with synthetic training data. *Remote Sensing of Environment, 246*, 111810. DOI: 10.1016/j.rse.2020.111810

6 Statistische Ämter des Bundes und der Länder. (2020). *Fläche und Bevölkerung nach Ländern*. Available: statistikportal.de/de/bevoelkerung/flaeche-und-bevoelkerung
